# Cuticular hydrocarbons as caste-linked cues in Neotropical swarm-founding wasps

**DOI:** 10.7717/peerj.13571

**Published:** 2022-06-07

**Authors:** Rafael Carvalho da Silva, Amanda Prato, Ivelize Tannure-Nascimento, Cintia Akemi Oi, Tom Wenseleers, Fabio Nascimento

**Affiliations:** 1 Departamento de Biologia/Faculdade de Filosofia Ciências e Letras de Ribeirão Preto, Universidade de São Paulo, Ribeirão Preto, São Paulo, Brazil; 2 Departamento de Morfologia e Fisiologia Animal, Universidade Estadual Paulista, Jaboticabal, São Paulo, Brazil; 3 Laboratory of Socioecology and Social Evolution, Katholieke Universiteit Leuven, Leuven, Belgium; 4 Centre for Biodiversity and Environment Research, University College London, University of London, London, United Kingdom

**Keywords:** Chemical signaling, Castes, Queen pheromones, Reproduction

## Abstract

Wasps (Vespidae) are important organisms to understand the evolution of social behaviour. Wasps show different levels of sociality, which includes solitary to highly eusocial organisms. In social insect species, queens and workers differ in physiology and morphology. The Neotropical swarm-founding wasps (Epiponini) show a variety of caste syndromes. In this clade, the caste-flexibility is a unique characteristic, in which workers can become queens and swarm to start a new nest. The investigation of the caste system comparing several Epiponini species show a clear-cut morphological distinction between queens and workers, with a morphological continuum between queens and workers. However, whether cuticular hydrocarbons (CHCs) are used as cues for caste recognition in swarm-founding wasps is still unknown. We studied whether CHCs may display caste-linked differences in eleven species of Epiponini wasps and if CHCs differences would follow morphological patterns. Our results suggest that queens and workers of Epiponini wasps are chemically different from each other at two levels, qualitatively and quantitatively, or merely quantitatively. This variation seems to exist regardless of their morphological traits and may be useful to help us understanding how chemical communication evolved differently in these species.

## Introduction

The evolution of reproductive castes is important for the organization of social insects (Queller & Strassmann, 1998). Among the hymenopteran social insects, the division of reproductive and nonreproductive labour was a fundamental step for their ecological success in most environments. In this context, Vespidae wasps are a key group for understanding the evolution of social behaviour because of their degrees of sociality, from solitary to highly eusocial (West-Eberhard, 1978; West-Eberhard, 1996; Wilson, 1985; Spradbery, 1991). They are important to understand the evolution of social behaviour as they are considered monophyletic, and a double origin of eusociality was recently supported in this group (Piekarski et al., 2018).

Within the subfamily Polistinae, the Neotropical tribe Epiponini (hence after epiponine) has perennial colonies and several inseminated breeders with a variable number of workers start new colonies (Jeanne, 1991; Jeanne, 2020). Epiponine and *Polistes* paper wasps are sister groups sharing a common caste-based ancestor (Menezes, Lloyd & Brady, 2020). It is then expected a conserved function of cuticular hydrocarbons (CHCs) in caste signalling and similarities of physiology and behaviour (Kelstrup et al., 2014a). The phenotype of reproductive individuals in several species of epiponine wasps is expressed by ovary activation (and insemination), which suggests post-imaginal caste determination (West-Eberhard, 1996), and retention of reproductive totipotence (Strassmann, Sullender & Queller, 2002; Mateus, Noll & Zucchi, 2004). For example, in *Parachartergus fraternus*, females with activated ovarioles play typical worker roles in their colonies (Mateus, Noll & Zucchi, 2004). However, in other species, caste fate seems to be determined by differential nourishment during larval development (Hunt et al., 1996).

During the colony cycle of epiponine, there is a reduction in the number of breeders (cyclical olygogyny) and workers evaluate queens’ fertility through aggressive solicitation, namely queen dance. In the queen dance, the queen reacts against workers. Then, submissive females bend their abdomen to perform a ritualized set of aggressive movements (West-Eberhard, 1978; Nascimento, Tannure-Nascimento & Zucchi, 2004; Kelstrup et al., 2014b). Yet, it is unknown if reproductive dominance is also pheromone-based in epiponine wasps, but past studies have shown that dominance signalling is likely to occur in two distinct epiponine species, *Polybia micans* and *Synoeca surinama* (Kelstrup et al., 2014a, 2014b). CHCs have been shown to function as queen pheromones across several lineages of social Hymenoptera (Holman et al., 2010; Holman, Lanfear & D’Ettorre, 2013; Oi et al., 2015; Oliveira et al., 2015; Van Oystaeyen et al., 2014; Holman et al., 2017). Most studies investigating CHCs as fertility cues in social insects are focused on ants (Holman, Lanfear & D’Ettorre, 2013; Van Oystaeyen et al., 2014; Monnin & Peeters, 1998; Liebig et al., 1999; Cuvillier-Hot et al., 2002), bees (Oliveira et al., 2015; Van Oystaeyen et al., 2014; Ayasse et al., 1995; Bloch & Hefetz, 1999; Andrade-Silva & Nascimento, 2015) and Polistinae or Vespinae wasps (Oi et al., 2016; Van Oystaeyen et al., 2014; Antonialli-Junior et al., 2021; da Silva et al., 2021; Sledge, Boscaro & Turillazzi, 2001; Sledge et al., 2004; Tannure-Nascimento, Nascimento & Zucchi, 2008; Oi et al., 2019). These studies demonstrated a correlation between the reproductive status of breeders and the presence of specific CHCs (Oi et al., 2015; Sledge et al., 2004; Tannure-Nascimento, Nascimento & Zucchi, 2008; Oi et al., 2019; Blomquist & Bagnères, 2010), and some of them evaluated the bioactivity of some CHCs (Oi et al., 2016; Van Oystaeyen et al., 2014; Holman et al., 2010). The correlative and empirical evidence support the hypothesis that the signals produced by reproductive females are likely honest messages of fertility rather than active inhibitors of workers’ reproduction (Keller & Nonacs, 1993; Beekman, 2004). For instance, in the common wasp *Vespula vulgaris*, two linear alkanes (*n*-C27 and *n*-C29) and one branched alkane (3-MeC29), which are present in relative higher quantities over the cuticular surface of queens, were found to reduce ovary activation in workers acting as queen pheromones (Van Oystaeyen et al., 2014). In the Saxon wasp, *Dolichovespula saxonica*, daily applications for two weeks of a mix of queen-characteristic CHCs (compounds overexpressed over the cuticular surface of queens), represented by three linear and two branched alkanes (*n*-C29, 3-MeC29, *n*-C30, *n*-C31, and 3-MeC31) resulted in reducing the proportion of workers with activated ovaries when compared with the solvent treated ones, meaning that one or more of the CHCs present in the tested blend, act as queen pheromones (Oi et al., 2016). Among the *Polistes* paper wasps, odd linear alkanes (from *n*-C_27_ to *n*-C_35_), methyl-branched alkanes (such as 7-MeC_27_; 7-MeC_33_; 13, 15, 17-C_33_), and some alkenes (C_27_:1, 9-C_29_:1, 9-C_31_:1, C_35_:2) seem to be candidates to signal reproductive status (Sledge, Boscaro & Turillazzi, 2001; Sledge et al., 2004; Tannure-Nascimento, Nascimento & Zucchi, 2008; Oi et al., 2019; Dapporto, Sledge & Turillazzi, 2005). Higher proportions of alkanes and dimethyl-alkanes were observed in breeders of hover wasps (Turillazzi et al., 2004). Among the few evidences available for *Mischocyttarus* wasp (Ferreira et al., 2022), two branched alkanes (8,12-diMeC32, and 6-MeC32) were found in higher proportions over the cuticular surface of queens (females engaged in reproductive tasks) and in females expressing ovary activation (but not necessarily involved in reproductive tasks), in *Mischocyttarus parallelogrammus* (da Silva et al., 2020a). In *Mischocyttarus cerberus*, 5-MeC29 and C33:1 were detected exclusively in samples of alpha females from pre-worker emergence nests, whereas in samples from post-worker emergent nests, a dimethyl and two methyl alkanes were found either exclusively or higher in proportion in alpha females (da Silva et al., 2020b). Finally, in the epiponine species, CHCs such as 3-MeC_25_ and *n*-C_25_ appeared to be queen-associated in *Polybia micans* (Kelstrup et al., 2014b), and the linear alkanes *n*-C18, *n*-C25, and *n*-C26 are more abundant in queens of *Polybia sericea* (Soares et al., 2021). In the warrior wasp, *Synoeca ilheensis* (previously called *Synoeca septentrionalis*) (Lopes & Menezes, 2017) three linear alkanes were also found in greatest abundances in queens of three unrelated colonies (*n*-C25, *n*-C29, and *n*-C31) (Santos et al., 2018), whereas older queens of its sister species, *Synoeca surinama*, a single alkene C_25_:1 was spotted as queen-characteristic (Kelstrup et al., 2014a).

Even though epiponine wasps contains about 250 species—distributed in 19 genera—which are almost exclusive to the Neotropical region (Carpenter, Kojima & Wenzel, 2000; Noll, 2013; Menezes, Lloyd & Brady, 2020), this tribe remains poorly explored concerning their chemical ecology. In the present work, we aimed to study the CHC diversity in eleven species from different genera and explore caste-linked differences. For that, we asked whether CHCs distinguish reproductive (queens) and non-reproductive (workers) individuals of epiponine wasps—including species with and without morphological caste differences. We hypothesized that CHCs are expressed differently between queens and workers, despite their morphological caste differences, similar to what is seen for other hymenopteran species, whether CHCs act as badges of caste in epiponine wasps. Additionally, we expected to find a clear chemical variation between queens and workers in species where reproductive females and non-reproductive females also differ in morphology, and a chemical discrete variation in species where morphological features are similar between queens and workers.

## Material and Methods

### Field sampling

Mature colonies of eleven epiponine species (a total of 388 wasps were collected) were sampled in four localities in Brazil (Table 1). The species *Agelaia pallipes*, *Apoica flavissima*, *Metapolybia docilis* and *Polybia paulista* were collected in Ribeirão Preto, São Paulo State, Brazil. The species *Brachygastra augusti*, *Chartergerllus communis*, *Nectarinella xavantinensis* and *Parachartergus fraternus* were collected in Nova Xavantina, Mato Grosso state, Brazil. The species *Charterginus* sp. and *Clypearia sulcata* were sampled in Novo Airão, Amazonas state, Brazil. *Synoeca surinama* was sampled in São Cristóvão, Sergipe state, Brazil. The comparisons between queens and workers were performed for females collected from the same nest. The classification of the eleven species in caste syndromes, according to Noll et al. (2020) is shown in Table 1. According to Noll et al. (2020) the caste syndromes consist of three different categories (I) all females are morphological similar, (II) reproductive females (queens) are isometrically larger (big versions of workers) than non-reproductive ones (workers), and (III) reproductive females (queens) allometrically different (they have some body structures that are disproportionally larger than in workers) from non-reproductive ones (workers). All females were frozen-killed and, then individually separated from the brood and kept in a freezer (−20 °C) for posterior CHCs extraction and dissection. To extract the CHCs, each female was added to a glass vial which was then filled with hexane for two minutes, then the bodies were removed from the glass vial and were preserved in a plastic tube containing alcohol 70% until the dissections, to check ovarian status and insemination.

**Table 1:** Collected species of epiponine wasps and morphological group syndromes.

Species	Locality	Year	Caste-syndrome group
*Chartergerllus communis*	Nova Xavantina - MT, Brazil	2010	Group I
*Metapolybia docilis*	Ribeirão Preto - SP, Brazil	2010	Group I
*Nectarinella xavantinensis*	Nova Xavantina - MT, Brazil	2010	Group I
*Synoeca surinama*	São Cristóvão - SE, Brazil	2013	Group I
*Parachartergus fraternus*	Nova Xavantina - MT, Brazil	2010	Group I
*Clypearia sulcata*	Novo Airão - AM, Brazil	2017	Group I
*Brachygastra augusti*	Nova Xavantina - MT, Brazil	2010	Group II
*Polybia paulista*	Ribeirão Preto - SP, Brazil	2016	Group II
*Charterginus* sp.	Novo Airão - AM, Brazil	2017	Group II
*Agelaia pallipes*	Ribeirão Preto - SP, Brazil	2010	Group III
*Apoica flavissima*	Ribeirão Preto - SP, Brazil	2010	Group III

**Note:**

Descriptive general features according to Noll et al. (2020): Group I, all females morphologically similar; Group II, reproductive females isometrically different from non-reproductive ones; Group III, reproductive females allometrically different from non-reproductive ones. Physiological features of *Chartergerllus communis*, *Nectarinella xavantinensis* and *Parachartergus fraternus*-all females can have activated ovaries; physiological features of *Metapolybia docilis*, *Synoeca surinama*, *Clypearia sulcata*, *Polybia paulista* and *Apoica flavissima*-only queens have activated ovaries; physiological features of *Brachygastra augusti*, *Charterginus* sp. and *Agelaia pallipes*-queens and a few females can have activated ovaries.

### Ovarian activation for caste assignment

**Figure 1:**
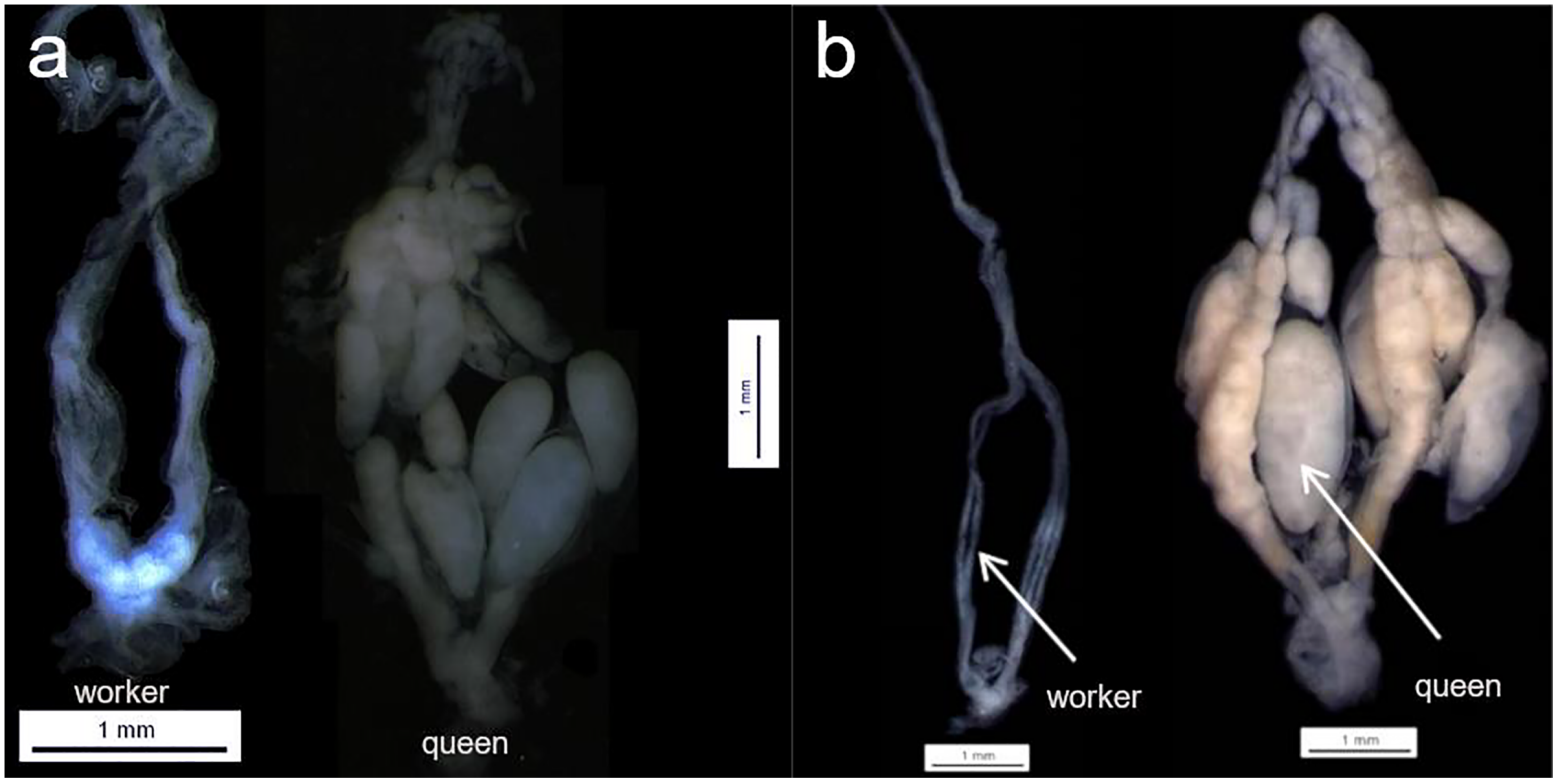
Queen and workers ovaries. Examples of queen worker ovaries found during the dissection section. (A) *Parachartergus fraternus*; (B) *Apoica flavissima*. White arrows are indicating the absence of oocytes (worker) and one mature of oocyte (queen) in *A. flavissima*.

Females were dissected and assigned to two different groups (i) queens, or (ii) workers based on their ovarian status. For the ovarian activity status, most of the females were dissected under a stereomicroscope Leica MZ75, however due to the difficult to sample rare species, some of the samples were dissected in the field after the sampling, for that, a LCD Digital microscope inskam-307 was used. Considering that not all species were collected at the same time, pictures are not available for all the eleven species (due to fieldwork constraints), however, the same parameters were adopted during the female assignment (see example Fig. 1). Ovaries were classified into two different categories without apparent activation—threadlike or filamentous ovarioles—(for workers), and with apparent activation—ovarioles with full mature oocytes—(for queens) (Fig. 1—The number of queens and workers per species is provided in Table S1). Only two categories of ovary activation were adopted in order to avoid misclassification. We were not able to check the spermatheca status for all the females; however, when possible, this information was used to confirm the group assignment (queen or worker together with their ovary status). To check whether the females were inseminated or not, the spermatheca was removed and prepared on a glass slide. The presence of yellow lines indicated that the spermatheca was inseminated, whereas the absence represented not inseminated.

### CHCs analysis

Glass vials containing CHCs were resuspended with 200 µl of hexane, and 2 µl was injected in the machine. The samples were analysed in a Gas Chromatography system coupled to SHIMADZU Mass Spectrometry (GC-MS), model GCMS-QP2010, equipped with a 25 m silicon capillary column (Rtx-5MS) and helium as carrier gas at 1 ml/min, using chemical ionization (CI) methods and electronic ionization (EI) to determine the peak molecular spectrum and provide information about the structures. Subsequently, the chromatograms were analysed to check the mass spectrum and identify the chemical compounds. Equivalent chain lengths were determined using standard n-alkanes (*n*-C_21_ to *n*-C_40_) (Sigma Chemical Co., St. Louis, MI, USA), and quantification was based on the peak areas obtained by the chromatograms (Singer, Camann & Espelie, 1992). Considering the samples were collected in different times and that we aimed to compare quantitative levels of CHCs across the castes, the adjustments in the temperature parameters for GC-MS analyses were different per species to optimize their chromatogram resolution (for specific methods see Table 2). Analyses were performed in splitless mode.

**Table 2:** GCMS parameters used in this study.

Species	Caste-syndrome group	IT (°C)	TD	FT
*Chartergerllus communis*	Group I	150	Increased to 280 °C - rate 10 °C/min	280 °C - held for 20 min
*Metapolybia docilis*	Group I	150	Increased to 280 °C - rate 10 °C/min	280 °C - held for 20 min
*Nectarinella xavantinenesis*	Group I	150	Increased to 280 °C - rate 10 °C/min	280 °C - held for 12 min
*Synoeca surinama*	Group I	150	Increased to 280 °C - rate 10 °C/min	280 °C - held for 20 min
*Parachartergus fraternus*	Group I	150	Increased to 280 °C - rate 10 °C/min	280 °C - held for 12 min
*Clypearia sulcata*	Group I	150	Increased to 270 °C - rate 5 °C/min; increased to 310 °C - rate 4 °C/ min; increased to 320 °C - rate 1 °C/min	320 °C - held for 10 min
*Brachygastra augusti*	Group II	150	Increased to 280 °C - rate 10 °C/min	280 °C - held for 20 min
*Polybia paulista*	Group II	180	Increased to 260 °C - rate 12 °C/min - held 5 min; increased to 300 °C - rate 5 °C/min	300 °C - held for 5 min
*Charterginus* sp.	Group II	180	Increased to 260 °C - rate 3 °C/min; increased to 270 °C - rate 1 °C/min; increased to 310 °C - rate 5 °C/min	310 °C - held for 10 min
*Agelaia pallipes*	Group III	150	Increased to 280 °C - rate 10 °C/min	280 °C - held for 20 min
*Apoica flavissima*	Group III	150	Increased to 300 °C - rate 5 °C/min	300 °C - held for 25 min

**Note:**

Specific parameters used to analyze cuticular hydrocarbons in the GC-MS for the eleven species. IT, initial temperature; TD, temperature during the analyse; FT, final temperature.

### Statistical analysis

Peak areas of each compound identified in the chromatograms of queens and workers of all studied species were transformed in relative amounts. First, we ran a permutation analysis (PERMANOVA) to check whether the two groups of females of all species (queen and workers) differed based on their chemical profiles, in addition to that, the same test was run separately considering only compounds per class (linear alkanes, methyl alkanes, dimethyl alkanes, trimethyl alkanes, alkenes and alkadienes). For this analysis we used the *adonis* function from the *vegan* package and 9,999 permutations were adopted (Oksanen et al., 2013). Finally, we run a multivariate similarity analysis (SIMPER) using the Bray-Curtis distance and adopting 999 permutations to check how much each chemical variable contributed to the observed differences between queens and workers. In the output of SIMPER, it is possible to access the average in which each CHC contribute to the average overall dissimilarity. For this analysis, we used the *simper* function from the *vegan* package (Oksanen et al., 2013). All statistical analyses were performed using R (version 4.0.2) (R Core Team, 2018). We also included the differentiation in relative amount per group of compounds, using linear alkanes, methyl-alkanes, dimethyl-alkanes, trimethyl-alkanes, alkenes and alkadienes.

Field experiments were approved by the Research Council of the Universidade de São Paulo (Process 2010/10027-5) and Sisbio #46555-5.

## Results

**Figure 2:**
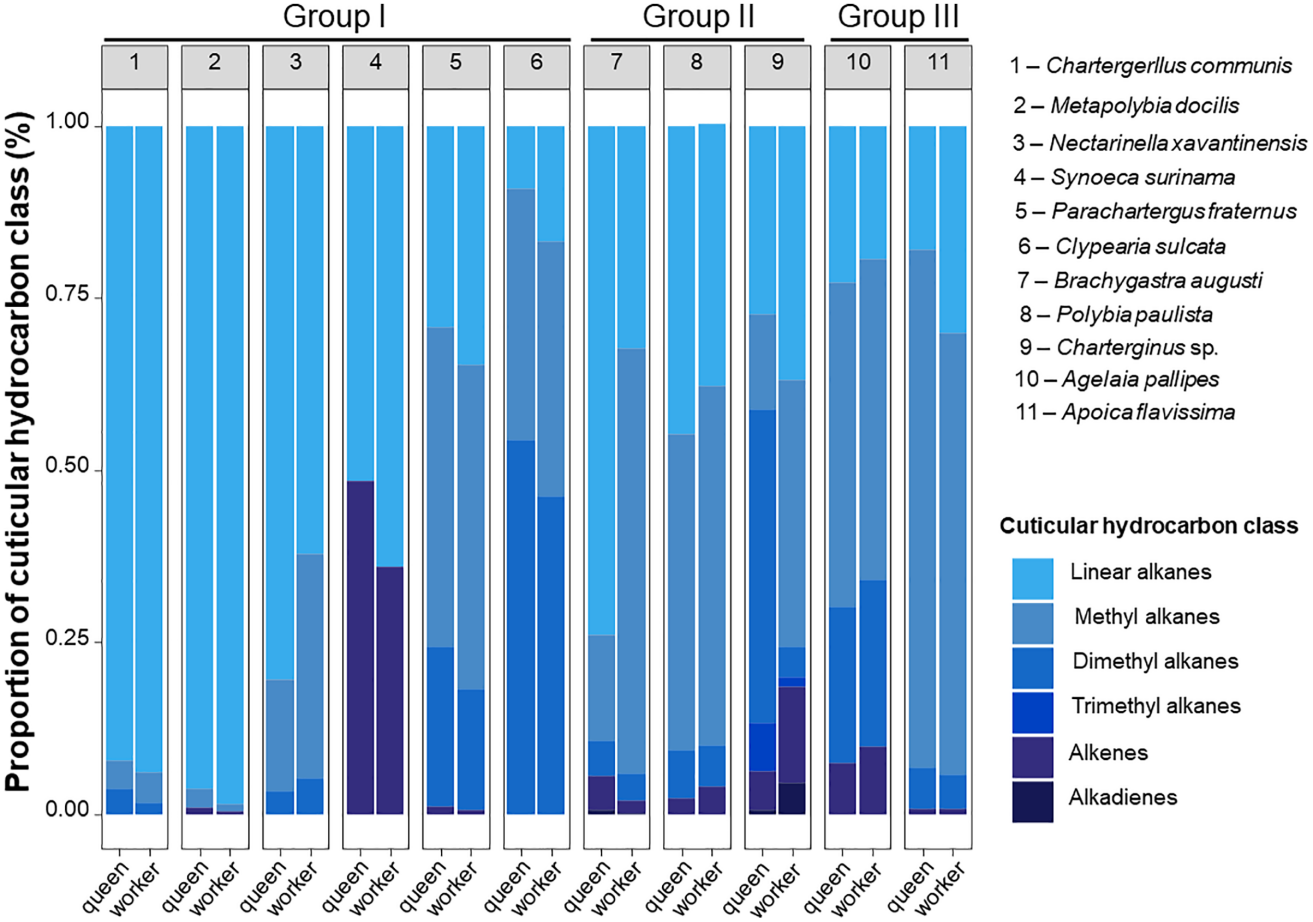
Schematic proportion of cuticular compounds found in queen and workers Epiponini species. Queens and workers of epiponine wasps express different of proportions of cuticular hydrocarbons (CHCs). Schematic representation of CHCs classes identified in the species of the present study. Each bar plot represents the average CHC composition expressed by queens and workers of the eleven studied species. Numbers within the grey boxes correspond to the study species. Group I, all females morphologically similar; Group II, reproductive females isometrically different from workers; Group III, reproductive females allometrically different from workers. The group classification is based on Noll et al. (2020).

The CHC analysis revealed 232 compounds in total, mostly hydrocarbons, which included linear alkanes, methyl-alkanes, dimethyl-alkanes, trimethyl-alkanes, alkenes and alkadienes (Table 3, Tables S1 and S2). These compounds ranged from 18 to 40 of carbon length. Only four compounds were common to all study species, *n*-C25, *n*-C27, *n*-C28 and *n*-C29. The species with the highest chemical diversity was *Charterginus* sp., which expressed 64 chemical compounds, while the species with the least chemical diversity was *N. xavantinensis* with 21 chemical compounds. The percentage of CHC classes expressed by queens and workers of each species are shown in Fig. 2, Table 3 and Table S2.

**Table 3:** Classes of cuticular hydrocarbons and wasp species.

C.S.	Species	Group	Linear alkanes	Methyl alkanes	Dimethyl alkanes	Trimethyl alkanes	Alkenes	Alkadienes
Group I	*Chartergerllus communis*	G	93.54	4.30	2.16	0.00	0.00	0.00
		Q	92.14	4.06	3.80	0.00	0.00	0.00
		W	93.92	4.37	1.71	0.00	0.00	0.00
	*Metapolybia docilis*	G	97.76	1.62	0.00	0.00	0.62	0.00
		Q	96.27	2.80	0.00	0.00	0.93	0.00
		W	98.40	1.11	0.00	0.00	0.49	0.00
	*Nectarinella xavantinensis*	G	65.56	29.54	4.90	0.00	0.00	0.00
		Q	80.39	16.27	3.34	0.00	0.00	0.00
		W	62.14	32.60	5.26	0.00	0.00	0.00
	*Synoeca surinama*	G	58.82	0.00	0.00	0.00	41.18	0.00
		Q	51.56	0.00	0.00	0.00	48.44	0.00
		W	63.99	0.00	0.00	0.00	36.01	0.00
	*Parachartergus fraternus*	G	32.60	46.74	19.78	0.00	0.88	0.00
		Q	29.30	46.28	23.32	0.00	1.10	0.00
		W	34.71	47.04	17.52	0.00	0.73	0.00
	*Clypearia sulcata*	G	15.48	37.06	47.46	0.00	0.00	0.00
		Q	9.03	36.60	54.37	0.00	0.00	0.00
		W	16.72	37.15	46.13	0.00	0.00	0.00
Group II	*Brachygastra augusti*	G	49.91	42.18	4.38	0.00	3.21	0.32
		Q	73.99	15.33	5.01	0.00	5.07	0.60
		W	32.25	61.88	3.92	0.00	1.85	0.10
	*Polybia paulista*	G	41.32	48.98	6.54	0.00	3.16	0.00
		Q	44.82	45.86	7.00	0.00	2.32	0.00
		W	37.81	52.10	6.09	0.00	4.00	0.00
	*Charterginus* sp.	G	35.10	33.89	12.36	2.53	12.32	3.80
		Q	27.29	13.94	45.44	7.10	5.54	0.69
		W	36.98	38.67	4.42	1.43	13.95	4.55
Group III	*Agelaia pallipes*	G	20.54	46.77	23.70	0.00	8.99	0.00
		Q	22.73	47.09	22.74	0.00	7.44	0.00
		W	19.36	46.60	24.22	0.00	9.82	0.00
	*Apoica flavissima*	G	24.48	69.28	5.38	0.00	0.86	0.00
		Q	18.05	75.17	5.98	0.00	0.80	0.00
		W	30.10	64.14	4.86	0.00	0.90	0.00

**Note:**

Percentage of cuticular hydrocarbon class found in samples of queens and workers of the eleven species of Epiponini wasps. C.S., Caste Syndrome; Group I, all females morphologically similar; Group II, reproductive females isometrically different from workers; Group III, reproductive females allometrically different from workers. G, general values per species; Q, values per queens; W, values per workers.

Furthermore, we performed the chemical analyses according to caste syndromes that are depicted below:

### All females morphologically similar

Queens and workers of *C. communis* have the same cuticular composition, with compounds ranging from 19 to 31 carbon lengths (Table S1). Permutation analysis revealed that queens and workers of *C. communis* are similar to each other in terms of chemical cuticular composition (Table 4). The ten most relevant compounds according to the SIMPER analysis included mostly linear alkanes and a few branched alkanes (Table 5). Two compounds were significantly different between queens and workers (*n*-C27 and *n*-C31) (Table 5). Queens and workers of *M. docilis* shared the same compounds, which ranged from CHCs with 20 to 33 carbon lengths (Table S1). According to permutation analysis, queens and workers of *M*. *docilis* do not differ based on their chemical profile composition (Table 4). The ten most important compounds according to SIMPER analysis included majority linear alkanes and one methyl-alkane (Table 5). Only one compound differed significantly between queens and workers (*n*-C24) (Table 5). Queens and workers of *N. xavantinensis* shared the same compounds, which ranged from CHCs with 24 to 31 carbon lengths (Table S1). Permutation analysis revealed that queens and workers of *N. xavantinensis* are not different based on their chemical composition (Table 4). The ten most relevant compounds according to SIMPER included mostly linear alkanes, followed by methyl-alkanes and one dimethyl-alkane (Table 5). Two compounds were significantly different between queens and workers (*n*-C29 and *n*-C31) (Table 5). Queens and workers of *S. surinama* have almost the same cuticular composition, with exception for n-C37, which was found in a low proportion in queens, their CHCs ranged from 21 to 37 carbon lengths (Table S1). According to permutation analysis, queens and workers of *S*. *surinama* can be distinguished based on their chemical cuticular composition (Table 4). The ten most relevant compounds according to SIMPER included linear alkanes and linear alkenes (Table 5). Almost all ten compounds differed significantly between queens and workers (C25:1-1, C31:1, *n*-C31, C33:1, *n*-C33, *n*-C27, *n*-C29, C27:1 and *n*-C23) (Table 5). Queens and workers of *P. fraternus* shared the same compounds, which ranged from CHCs with 18 to 30 carbon lengths (Table S1). Queens and workers of *P. fraternus* are chemically distinct according to permutation analysis (Table 4). The ten most relevant compounds according to SIMPER included linear and branched alkanes (Table 5). Of this, six were significantly different between queens and workers (the mixture of 3,11-; 7,9-diMeC29 and *n*-C30, 11,13-diMeC29, 13,17-diMeC28, 5-MeC25, 9-MeC25 and *n*-C25) (Table 5). Lastly, queens and workers of *C. sulcata* did not share the same CHCs. Their compounds consisted of CHCs having 23 to 39 carbon lengths (Table S1). Queens and workers of *C*. *sulcata* are different from each other in terms of chemical composition according to permutation analysis (Table 4). The ten most important compounds according to SIMPER included mostly dimethyl-alkanes, but also methyl-alkanes and linear alkanes (Table 5). Five compounds were significantly different between queens and workers (17-;15-;13-;11-;9-;7-MeC35, *n*-C31, 13,17-diMeC33, 7,11-diMeC35 and x,y-diMeC37) (Table 5).

**Table 4:** Permutation analysis of cuticular hydrocarbons.

C.S.	Species	Groups	F value	R²	*p* value
Group I	*Chartergerllus communis*	All compounds	1.995	0.142	0.087
		Linear alkanes	2.344	0.163	0.072
		Methyl alkanes	0.811	0.063	0.535
		Dimethyl alkanes	1.699	0.124	0.177
	*Metapolybia docilis*	All compounds	1.481	0.156	0.249
		Linear alkanes	1.516	0.159	0.248
		Methyl alkanes	1.108	0.121	0.312
		Alkenes	0.567	0.066	0.711
	*Nectarinella xavantinensis*	All compounds	3.024	0.177	0.072
		Linear alkanes	2.597	0.156	0.078
		Methyl alkanes	2.49	0.151	0.098
		Dimethyl alkanes	0.836	0.056	0.408
	*Synoeca surinama*	All compounds	105.65	0.399	**0.0001**
		Linear alkanes	57.593	0.265	**0.0001**
		Alkenes	167.53	0.513	**0.0001**
	*Parachartergus fraternus*	All compounds	6.133	0.277	**0.0003**
		Linear alkanes	4.235	0.209	**0.011**
		Methyl alkanes	5.757	0.264	**0.0002**
		Dimethyl alkanes	9.238	0.366	**0.001**
		Alkenes	5.909	0.269	**0.005**
	*Clypearia sulcata*	All compounds	7.634	0.208	**0.001**
		Linear alkanes	10.241	0.26	**0.0001**
		Methyl alkanes	4.462	0.133	**0.024**
		Dimethyl alkanes	7.897	0.214	**0.002**
Group II	*Brachygastra augusti*	All compounds	22.043	0.478	**0.0001**
		Linear alkanes	16.989	0.414	**0.0001**
		Methyl alkanes	15.352	0.39	**0.0001**
		Dimethyl alkanes	13.611	0.361	**0.0001**
		Alkenes	10.387	0.302	**0.0008**
		Alkadienes	–	–	–
	*Polybia paulista*	All compounds	6.764	0.183	**0.0013**
		Linear alkanes	10.092	0.251	**0.0005**
		Methyl alkanes	2.66	0.081	**0.07**
		Dimethyl alkanes	7.089	0.191	**0.0007**
		Alkenes	4.595	0.132	**0.0004**
	*Charterginus* sp.	All compounds	62.517	0.683	**0.0001**
		Linear alkanes	12.866	0.307	**0.0001**
		Methyl alkanes	52.962	0.646	**0.0001**
		Dimethyl alkanes	98.247	0.772	**0.0001**
		Trimethyl alkanes	–	–	–
		Alkenes	85.595	0.746	**0.0001**
Group III	*Agelaia pallipes*	All compounds	9.129	0.221	**0.0001**
		Linear alkanes	9.721	0.233	**0.0001**
		Methyl alkanes	8.01	0.2	**0.0001**
		Dimethyl alkanes	7.817	0.196	**0.0004**
		Alkenes	12.558	0.281	**0.0001**
	*Apoica flavissima*	All compounds	3.277	0.201	0.052
		Linear alkanes	3.596	0.216	**0.044**
		Methyl alkanes	2.689	0.171	0.054
		Dimethyl alkanes	3.442	0.209	**0.049**
		Alkenes	0.543	0.04	0.683

**Note:**

Comparisons between queens and workers considering all compounds and compound classes separately. C.S, caste-syndrome group. Degrees of freedom equals to 1.

Bold numbers are significant values. Italics denote wasp species.

**Table 5:** Most important contributor compounds to separate queens and workers of epiponine wasps.

	Average	Sd	Ratio	Av. worker	Av. queen	Cum. sum	*p*-value	Sig. level
(a) *Chartergerllus communis*								
n-C29	0.032	0.021	1.532	32.613	36.935	0.202	0.538	n.s
n-C27	0.028	0.016	1.790	44.290	38.949	0.379	0.032	*
n-C30	0.014	0.020	0.705	1.540	3.203	0.471	0.514	n.s
n-C25	0.013	0.009	1.486	3.637	2.103	0.555	0.215	n.s
n-C31	0.011	0.007	1.595	3.967	6.003	0.627	0.046	*
n-C28	0.008	0.005	1.606	2.933	3.536	0.679	0.604	n.s
15-;13-;11-MeC29	0.008	0.006	1.257	1.453	1.206	0.729	0.469	n.s
9,13-diMeC24	0.007	0.005	1.293	1.590	0.561	0.773	0.057	n.s
11,13-diMeC27	0.005	0.006	0.829	1.083	0.249	0.803	0.084	n.s
13-;11-MeC27	0.004	0.005	0.800	0.767	0.151	0.826	0.057	n.s
(b) *Metapolybia docilis*								
n-C31	0.044	0.035	1.270	30.340	38.664	0.287	0.311	n.s
n-C29	0.036	0.025	1.430	44.240	44.079	0.519	0.678	n.s
n-C27	0.021	0.016	1.304	6.587	3.454	0.658	0.098	n.s
n-C30	0.006	0.004	1.620	3.947	3.137	0.696	0.571	n.s
n-C28	0.006	0.003	1.989	2.923	2.050	0.733	0.324	n.s
n-C32	0.005	0.004	1.332	1.713	1.134	0.767	0.597	n.s
n-C24	0.005	0.003	1.470	1.683	0.814	0.797	0.033	*
n-C25	0.004	0.005	0.760	0.337	0.896	0.821	0.917	n.s
15-;13-MeC27	0.004	0.004	0.866	0.773	0.219	0.844	0.243	n.s
n-C33	0.003	0.004	0.929	1.090	1.387	0.865	0.824	n.s
(c) *Nectarinella xavantinenis*								
n-C29	0.084	0.054	1.573	28.100	13.315	0.238	0.019	*
n-C25	0.058	0.033	1.722	20.990	25.521	0.400	0.221	n.s
n-C31	0.040	0.022	1.830	11.203	3.178	0.514	0.003	**
15-;13-MeC29	0.038	0.025	1.514	5.727	11.944	0.619	0.344	n.s
15-;13-MeC27	0.036	0.024	1.490	4.720	11.468	0.719	0.091	n.s
n-C27	0.029	0.020	1.448	14.617	15.723	0.800	0.982	n.s
15,17-diMeC29	0.015	0.013	1.198	2.400	4.650	0.843	0.863	n.s
11-;9-MeC27	0.010	0.008	1.262	2.207	3.488	0.871	0.877	n.s
n-C24	0.007	0.006	1.109	1.563	1.118	0.890	0.220	n.s
13-;11-MeC25	0.005	0.004	1.214	0.743	1.735	0.904	0.343	n.s
(d) *Synoeca surinama*								
C25:1-1	0.059	0.030	1.994	16.919	5.596	0.211	0.001	***
C31:1	0.037	0.021	1.745	23.972	16.593	0.345	0.001	***
n-C31	0.035	0.023	1.504	20.710	19.224	0.469	0.005	**
n-C25	0.026	0.023	1.141	13.108	14.316	0.563	1.000	n.s
C33:1	0.022	0.009	2.397	0.761	5.167	0.642	0.001	***
n-C33	0.022	0.011	1.978	1.598	5.956	0.719	0.001	***
n-C27	0.016	0.008	2.030	4.214	7.263	0.775	0.001	***
n-C29	0.010	0.007	1.334	9.199	8.029	0.809	0.001	***
C27:1	0.009	0.008	1.196	2.868	4.336	0.841	0.001	***
n-C23	0.007	0.007	1.081	0.318	1.704	0.866	0.001	***
(e) *Parachartergus fraternus*								
n-C27	0.035	0.039	0.885	14.574	21.112	0.155	0.268	n.s
15-;13-;11-;9-MeC27	0.020	0.015	1.375	19.657	18.198	0.247	0.841	n.s
n-C29	0.017	0.012	1.385	8.961	7.241	0.323	0.171	n.s
3,11-; 7,9-diMeC29 + n.C30	0.014	0.006	2.416	4.976	2.258	0.383	0.001	***
11,13-diMeC29	0.012	0.008	1.615	8.284	5.826	0.438	0.004	**
12-;10-;8-MeC28	0.012	0.011	1.043	11.270	9.345	0.491	0.364	n.s
13,17-diMeC28	0.010	0.006	1.739	4.813	2.835	0.536	0.001	***
5-MeC25	0.010	0.005	1.791	1.439	3.387	0.579	0.004	**
9-MeC25	0.009	0.005	1.821	1.227	2.956	0.620	0.012	*
n-C25	0.008	0.006	1.416	0.671	2.306	0.658	0.013	*
(f) *Clypearia sulcata*								
15,19-; 13,7-diMeC35	0.026	0.019	1.417	18.625	23.908	0.126	0.072	n.s
17-;15-;13-;11-;9-;7-MeC35	0.022	0.009	2.394	15.370	19.862	0.233	0.001	***
n-C31	0.019	0.010	1.813	5.798	2.160	0.322	0.013	*
13,21-;13,19-;11,15-diMeC37	0.015	0.009	1.590	10.904	8.882	0.392	0.438	n.s
13,17-diMeC33	0.013	0.006	2.397	3.950	6.594	0.455	0.001	***
n-C29	0.010	0.009	1.170	6.715	5.776	0.503	0.646	n.s
7,11-diMeC35	0.008	0.001	11.915	0.000	1.604	0.542	0.001	***
x,y-diMeC37	0.008	0.004	2.162	1.593	0.000	0.580	0.001	***
15-;13-;11-;9-MeC31	0.008	0.014	0.569	1.926	0.602	0.617	0.999	n.s
15-;13-;11-;9-;7-MeC37	0.008	0.005	1.470	10.168	8.922	0.654	0.215	n.s
(g) *Brachygastra augusti*								
n-C29	0.098	0.062	1.562	27.995	9.434	0.158	0.001	***
15-;13-;11-MeC31	0.094	0.035	2.693	1.319	19.967	0.310	0.001	***
15-;13-MeC33	0.066	0.028	2.379	2.550	15.597	0.418	0.001	***
13-;11-MeC29	0.057	0.022	2.544	1.557	12.950	0.511	0.001	***
n-C27	0.054	0.030	1.791	16.252	8.273	0.599	0.001	***
n-C31	0.039	0.049	0.783	10.605	4.064	0.662	0.001	***
13-MeC35	0.023	0.011	2.102	0.981	5.371	0.699	0.001	***
C29:1-1	0.019	0.021	0.895	3.216	1.239	0.729	0.063	n.s
5,11-diMeC30	0.017	0.018	0.986	3.124	1.038	0.757	0.038	*
17-;16-;14-;11-MeC34	0.016	0.013	1.207	2.781	2.186	0.783	0.002	**
(h) *Polybia paulista*								
n-C29	0.065	0.037	1.755	20.967	9.590	0.235	0.001	***
15-;13-;11-MeC29	0.030	0.020	1.498	22.563	23.836	0.343	0.142	n.s
n-C27	0.022	0.026	0.853	16.988	16.171	0.424	0.038	*
13-;11-MeC27	0.021	0.014	1.538	6.429	9.369	0.501	0.006	**
n-C25	0.016	0.011	1.374	1.884	4.631	0.558	0.001	***
n-C23	0.014	0.030	0.458	0.221	2.894	0.608	0.002	**
3-MeC27	0.011	0.008	1.333	5.036	5.166	0.647	0.284	n.s
C31:1	0.009	0.015	0.593	0.245	1.748	0.679	0.040	*
n-C31	0.009	0.005	1.772	2.444	1.414	0.711	0.006	**
C27:1	0.008	0.016	0.515	0.773	1.124	0.740	0.600	n.s
(i) *Charterginus* sp.								
11,21-;11,19-diMeC39	0.114	0.008	15.145	1.903	24.688	0.196	0.001	***
11,21-;11,19-diMeC37	0.049	0.010	5.085	1.427	11.160	0.279	0.001	***
C29:1-1	0.036	0.008	4.601	8.296	1.185	0.341	0.001	***
n-C29	0.034	0.016	2.146	9.772	3.020	0.399	0.001	***
3-MeC27	0.031	0.007	4.115	7.806	1.668	0.451	0.001	***
n-C27	0.021	0.019	1.103	18.390	15.195	0.488	0.682	n.s
5,19-;5,17-diMeC39	0.020	0.001	19.728	0.000	3.955	0.522	0.001	***
9-MeC27	0.017	0.008	2.070	3.860	0.408	0.552	0.001	***
15-;13-;11-MeC29	0.016	0.008	2.037	3.640	0.465	0.579	0.001	***
3-MeC29	0.014	0.004	3.205	3.234	0.520	0.602	0.001	***
(j) *Agelaia pallipes*								
x,y-diMeC28	0.015	0.012	1.291	6.785	4.421	0.086	0.181	n.s
15-;13-;11-MeC33	0.010	0.007	1.434	11.346	11.083	0.147	0.998	n.s
n-C29	0.010	0.009	1.104	8.190	8.242	0.204	1.000	n.s
n-C30	0.009	0.003	3.030	1.772	3.665	0.259	0.001	***
15-;13-;11-;9-;.7-MeC31	0.009	0.007	1.306	16.766	15.819	0.312	0.531	n.s
13,17-;11,15-diMeC33	0.008	0.005	1.506	7.037	7.378	0.359	0.970	n.s
17-;15-;13-;11-MeC35	0.008	0.008	0.954	2.680	3.085	0.404	0.431	n.s
15-;13-;11-;9-MeC29	0.007	0.009	0.822	6.692	6.382	0.444	0.966	n.s
13,17-;11,15-diMeC31	0.006	0.005	1.382	4.545	5.139	0.481	0.499	n.s
13,19-;13,17-diMeC35	0.006	0.004	1.512	2.727	3.212	0.514	0.579	n.s
(k) *Apoica flavissima*								
n-C27	0.039	0.042	0.931	10.479	17.139	0.206	0.243	n.s
11-MeC40	0.034	0.031	1.097	31.541	26.364	0.386	0.268	n.s
n-C29	0.026	0.026	0.991	4.836	9.564	0.521	0.106	n.s
17-;13-;11-MeC38	0.024	0.020	1.176	17.564	14.459	0.646	0.376	n.s
15-;13-;11-MeC31	0.010	0.010	0.988	9.291	7.716	0.700	0.357	n.s
13-MeC40	0.009	0.006	1.564	2.097	3.763	0.747	0.025	*
3-MeC29	0.009	0.004	2.409	3.561	2.505	0.792	0.037	*
13,15-;11,13-diMeC33	0.004	0.004	1.189	3.017	2.248	0.815	0.064	n.s
15-;13-;11-MeC29	0.004	0.003	1.147	1.599	1.465	0.834	0.949	n.s
17-;15-;13-MeC33	0.003	0.004	0.891	3.764	3.186	0.851	0.246	n.s

**Notes:**

Contribution of the first ten most important compounds discriminating queens and workers using SIMPER Bray-Curtis dissimilarities (999 permutations). (a) *Chartergerllus communis*; (b) *Metapolybia docilis*; (c) *Nectarinella xavantinensis*; (d) *Synoeca surinama*; (e) *Parachartergus fraternus*; (f) *Clypearia sulcata*; (g) *Brachygastra augusti*; (h) *Polybia paulista*; (i) *Charterginus* sp.; (j) *Agelaia pallipes*; (k) *Apoica flavissima*. sd, standard deviation; av. worker, compound average present in workers; av. queens, compound average present in queens; cum. sum, cumulative contribution (%) of each compound for differentiation; sig. level, significance level (n.s., non-significant).

* *p* < 0.05.

** *p* < 0.01.

*** *p* < 0.001.

### Reproductive females isometrically different from workers

Queens and workers of *B. augusti* express the same CHC composition, which includes compounds ranging from 20 to 35 carbon lengths (Tables S1 and S2). According to permutation analysis, queens and workers of *B. augusti* are chemically different (Table 4). The ten most important compounds according to SIMPER analysis comprised mostly methyl-alkanes, but also linear alkanes, one linear alkene and one dimethyl-alkane (Table 5). Nine out of 10 compounds were significantly different between queens and workers (*n*-C29, 15-;13-;11-MeC31, 15-;14-;13-MeC33, 13-;11-,MeC29, *n*-C27, *n*-C31, 13-MeC35, 5,11-diMeC30 and 17-;16-;14-;11-MeC34) (Table 5). Queens and workers of *P. paulista* shared almost the same CHCs, with only a few divergences (Table S1), the cuticular profiles included CHCs ranging from 22 to 33 carbon lengths. Queens and workers of *P*. *paulista* are chemically distinct (Table 5). The ten most relevant compounds according to SIMPER included mostly linear alkanes, but also linear alkenes and methyl-alkanes (Table 5). Seven compounds were significantly different between queens and workers (*n*-C29, *n*-C27, 13-;11-MeC27, *n*-C25, *n*-C23, C31:1 and *n*-C31) (Table 5). Finally, according to the permutation analysis, queens and workers of *Charterginus* sp. can be distinguished based on their chemical profiles (Table 4). Queens and workers of *Charterginus* sp. do not share the exact same CHCs (Table S1), their chemical profiles included CHCs ranging from 23 to 39 carbon lengths. According to SIMPER analysis the ten most relevant compounds responsible for promoting group differentiation included linear alkanes, methyl-alkanes, dimethyl-alkanes and one linear alkene (Table 5). Almost all compounds highlighted as the most relevant indeed differed significantly between queens and workers (11,21-; 11,19-diMeC39, 11,21-; 11,19-diMeC37, C29:1-1, *n*-C29, 3-MeC27, 5,19-; 5,17-diMeC39, 9-MeC27, 15-;13-;11-MeC29 and 3-MeC29) (Table 5).

### Reproductive females allometrically different from workers

Queens and workers of *A. pallipes* express almost the same CHCs, with only a few exceptions (Table S1), their chemical profiles include CHCs ranging from 21 to 35 carbon lengths. Queens and workers of *A*. *pallipes* can be chemically distinguished according to permutation analysis (Table 4). The ten most important compounds according to SIMPER included methyl and dimethyl-alkanes, and also linear alkanes (Table 5). A single compound was significantly different between queens and workers (*n*-C30) (Table 5). We found that queens and workers of *A. flavissima* express the same CHC profiles, which includes compounds ranging from 23 to 40 carbon lengths (Table S1). Lastly, according to permutation analysis, queens and workers of *A*. *flavissima* are not chemically distinct (Table 4). The ten most relevant compounds according to SIMPER included mostly methyl-alkanes, but also linear alkanes and one dimethyl-alkane (Table 5). Only two compounds were indeed different between queens and workers (13-MeC40 and 3-MeC29) (Table 5).

In five species (*C*. *sulcata*, *P. paulista*, *Charterginus* sp., *A*. *pallipes* and *A*. *pallens*) queens and workers differed both qualitatively and quantitively in terms of their CHC composition. We summarized which CHCs were present and absent in either of the castes (Table S2).

## Discussion

Overall, we confirmed our initial hypothesis that females belonging to different castes (queens and workers) express CHCs differently in epiponine wasps, seven out of eleven analyzed species were statistically different based on their overall chemical composition. Queens and workers expressed some caste specific CHCs (qualitative variation) in addition to different proportions of the shared compounds (quantitative variation), whereas in some other species the variation between queens and workers was subtle, consisting solely of quantitative variation. Contrary to our expectation, in species where females lack differential morphological traits, CHCs were either highly divergent but also similar between queens and workers depending on the species. As expected, species where females are morphologically distinct in caste, CHCs were majority distinct between queens and workers (with exception of *A*. *pallipes*). In this sense, CHCs are potential cues linked with caste in epiponine wasps.

CHCs have been demonstrated to be tightly connected with female fertility, and thus they are the candidates to act as queen pheromones in several unrelated lineages of social insects (Holman et al., 2010; Oliveira et al., 2015; Van Oystaeyen et al., 2014; Holman, 2018; Funaro et al., 2018; Layton, Camann & Espelie, 1994; Oliveira et al., 2017; Oi et al., 2016). In the case of epiponine queens, we found that in some species queens expressed exclusively certain CHCs, whereas in the remaining species, queens at least upregulate some of the CHCs that they share with workers. Interestingly, we reported four linear alkanes (*n*-C25, *n*-C27, *n*-C28, and *n*-C29) shared by females of all studied species, and overall, they were more abundant in the cuticular surface of queens rather than in workers. Considering the known properties of such compounds in advanced species of social wasps (*V*. *vulgaris* and *D*. *saxonica*) (Oi, 2022; Oi et al., 2016; Van Oystaeyen et al., 2014), these CHCs could potentially work as queen pheromones in swarm-founding species. From the literature, studies in epiponine wasps have revealed some specific compounds to be associated to queens. The results reported for *S. ilheensis* revealed that the overall proportion of linear alkanes was higher in queens compared to workers and males (Santos et al., 2018). In addition to that, three linear alkanes *n*-C25, *n*-C29, and *n*-C31, were consistently overexpressed by queens (Santos et al., 2018). In the species *P*. *sericea*, qualitative and quantitative variation were found between queens and workers, including females with and without ovary activation (Soares et al., 2021). For instance, the CHCs 3-MeC18, 9-MeC21, 14-;12-;10-MeC28, and *n*-C29 were found only in queens and workers with activated ovaries, whereas *n*-C20, 3-MeC25, and 2-MeC26 were identified exclusively in females with inactivated ovaries (Soares et al., 2021). Similarly, in *P. micans*, the linear alkane *n*-C25 and the branched alkane 3-MeC25, were significantly higher in queens (Kelstrup et al., 2014b). For now, the indication of putative queen pheromones in epiponine wasps is merely speculative and bioassays using synthetic versions of these chemicals should be conducted to test this hypothesis that linear alkanes act as queen pheromones in the swarm-founding wasps.

In epiponine wasps, it is still unknown whether the juvenile hormone (JH) plays a role regulating both queen fertility and queen signal production as it was shown in Vespinae wasps (Oliveira et al., 2017; Oi et al., 2021a; Oi et al., 2020). JH acts as a gonadotropic hormone and modulates CHCs expression in *S*. *surinama* (Kelstrup et al., 2014a), however, in *Polybia micans* (Kelstrup et al., 2014b), although it seems to control ovary activation, it does not have any correlation with CHCs expression. In *Polybia occidentalis*, JH regulates multiple factors in workers, such as age polyethism, ovarian status and CHCs expression (Prato et al., 2021). It was recently demonstrated that JH may also control female fertility and subtle modifications in CHCs in the primitively eusocial wasps *Polistes* and *Mischocyttarus* genera (Oi et al., 2021b). For the most part of our study species, epiponine queens were chemically distinguished from workers, it is possible that JH also controls CHCs production in these swarm-founding wasps, however, empirical evidence is needed. A systematic effort including species from different social insect taxa would be important to establish whether JH has a conserved function across the epiponine wasps.

We found a high chemical diversity among the epiponine wasp species, and each of them expressed a higher or lower CHC diversity. Even though queens and workers share similar proportions of CHCs classes, a species-specific qualitative variation was evident when the species were compared in Fig. 2. The chemical profiles of three species belonging to the Group I were majority composed by linear alkanes (*C*. *communis*, *M*. *docilis* and *N*. *xavantinensis*), while for *S*. *surinama* linear alkanes and alkenes were represented at similar proportional levels between castes, with queens being expressing more alkenes and workers linear alkanes, which is in line with the results previously published for the species (Kelstrup et al., 2014a). For the species *P*. *fraternus* and *C*. *sulcata*, which also belong to Group I, chemical profiles were more represented by branched alkanes. Females from Group II represented the ones with the most diversity in compound classes (*Charterginus* sp. = 6; *B*. *augusti* = 5; *P*. *paulista* = 4). Queens of *Charterginus* sp. exhibit branched alkanes in higher proportions whether compared to workers, which express linear and branched alkanes in similar proportions, on the other hand, in *B*. *augusti* the opposite pattern was seen, with queens being more represented by linear and workers by branched alkanes. Queens and workers of *P*. *paulista* show similar proportions of all compound classes. Lastly, females from both species comprising Group III were mainly characterized by branched alkanes (mostly methyl alkanes). More than 50% of the identified compounds comprise branched alkanes (methyl and dimethyl) and in a specific case (*S*. *surinama*), a high diversity of alkenes was found. Future studies could use CHCs as chemotaxonomic markers in epiponine, as most of our study species show a unique chemical signature. The use of CHCs as chemotaxonomic markers is common in the Hymenoptera order (Kather & Martin, 2015; Martin & Drijfhout, 2009; Dos Santos & Do Nascimento, 2015; Pokorny et al., 2014), solitary insects from Diptera (Ye et al., 2007; Moore et al., 2021), Hemiptera (Gemeno et al., 2012), and Orthoptera (Chapman, Espelie & Sword, 1995). For example, the chemical traits could also have a similar phylogenetic value compared to other often used characters, such as morphological, genetic or behavioural to build relationships among epiponine wasps.

## Conclusion

In conclusion, we demonstrated that queens and workers of epiponine wasps are chemically distinct based on their CHC profiles—and this dissimilarity exists regardless of their caste-syndromes—which includes qualitative and quantitative variation, but also only quantitative differences. To date, considering that Neotropical swarm-founding wasps remained less explored whether compared with other groups of social insects, the results presented here reinforce that CHCs may act as reliable cues of castes in different unrelated groups of social insects. To strengthen the understanding of their chemical variation, future studies need to study the CHCs dynamics of queens and workers on a broader scale and a higher diversity of species, controlling the age of the sampled females. Overall, our correlative results suggest that division of labour within epiponine societies may be mediated by CHCs, queens and workers may benefit from these chemical dissimilarities and use this information to maintain their colonies functioning cohesively.

## Supplemental Information

10.7717/peerj.13571/supp-1 Supplemental Information 1 Cuticular chemical composition of studied epiponine wasp species. Relative contribution (%) of cuticular hydrocarbon composition in Epiponini wasps from the present study. In parenthesis is presented the number of individuals analyzed per group from each species. sd = standard deviation. Considering that we did not perform the derivatization of the chemical samples, we could not confirm the right position of the double bounds for the alkenes identified. Thus, each of them was represented as unique in the table.

10.7717/peerj.13571/supp-2 Supplemental Information 2 Cuticular hydrocarbons (CHCs) that were found exclusively in queens or workers in the epiponine wasp species. Queens and workers of the remaining species shared the same CHC composition.

## Additional Information and Declarations

### Field Study Permissions

The following information was supplied relating to field study approvals (*i.e*., approving body and any reference numbers): Field experiments were approved by the Research Council of the Universidade de São Paulo (Processo 2010/10027-5).

### Data Availability

The following information was supplied regarding data availability: The raw data are available in the Supplemental File.
