# Supplementary material for: Cuticular hydrocarbons as caste-linked cues in Neotropical swarm-founding wasps
Source: PeerJ. 2022 Jun 7;10:e13571. doi: 10.7717/peerj.13571 (PMC9186331; doi:10.7717/peerj.13571)
Supplement: Supplemental Information 1 — Relative contribution (%) of cuticular hydrocarbon composition in Epiponini wasps from the present study. In parenthesis is presented the number of individuals analyzed per group from each species. sd = standard deviation. Considering that we did not perform the derivatization of the chemical samples, we could not confirm the right position of the double bounds for the alkenes identified. Thus, each of them was represented as unique in the table. [file peerj-10-13571-s001.docx]

Title: Cuticular hydrocarbons and caste-linked compounds in neotropical swarm-founding wasps

Authors: Rafael C. da Silva¹, Amanda Prato¹, Ivelize C. Tannure-Nascimento¹, Cintia A. Oi², Tom Wenseleers², Fábio S. Nascimento¹*

Affiliations: ¹ Faculdade de Filosofia, Ciências e Letras de Ribeirão Preto, Departamento de Biologia, Universidade de São Paulo – USP, Avenida Bandeirantes, 3900 – Vila Monte Alegre, Ribeirão Preto, SP 14040-900, Brazil ² Laboratory of Socioecology and Social Evolution, KU Leuven – University of Leuven, Naamsestraat 59 3000 Leuven, Belgium

*Corresponding author: Fábio S. Nascimento ([fsnascim@usp.br](mailto:fsnascim@usp.br))

**Supplementary information**

Table S1: Relative contribution (%) of cuticular hydrocarbon composition in Epiponini wasps from the present study. In parenthesis is presented the number of individuals analyzed per group from each species. sd = standard deviation. Considering that we did not perform the derivatization of the chemical samples, we could not confirm the right position of the double bounds for the alkenes identified. Thus, each of them was represented as unique in the table.

| caste syndromes | (I) Females morphologically similar | | | | | | | | | | | |
| --- | --- | --- | --- | --- | --- | --- | --- | --- | --- | --- | --- | --- |
| species | *Nectarinella xavantinensis* | | *Parachartergus fraternus* | | *Metapolybia docilis* | | *Chartergellus communis* | | *Synoeca surinama* | | *Clypearia sulcata* | |
| Compounds | Queens (3) | Workers (13) | Queens (7) | Workers (11) | Queens (3) | Workers (7) | Queens (3) | Workers (11) | Queens (67) | Workers (94) | Queens (5) | Workers (26) |
|  | mean±sd | mean±sd | mean±sd | mean±sd | mean±sd | mean±sd | mean±sd | mean±sd | mean±sd | mean±sd | mean±sd | mean±sd |
| n-C18 | - | - | 0.33±0.25 | 0.17±0.15 | - | - | - | - | - | - | - | - |
| n-C19 | - | - | - | - | - | - | 0.07±0.06 | 0.05±0.04 | - | - | - | - |
| n-C20 | - | - | - | - | 0.66±0.16 | 0.51±0.32 | 0.34±0.12 | 0.33±0.47 | - | - | - | - |
| n-C21 | - | - | 0.72±0.37 | 0.42±0.26 | 0.08±0.04 | 0.15±0.20 | 0.25±0.10 | 0.16±0.13 | 0.08±0.04 | 0.12±0.10 | - | - |
| n-C22 | - | - | 0.38±0.30 | 0.25±0.15 | 0.99±0.30 | 0.61±0.38 | 0.66±0.39 | 0.59±0.57 | 0.06±0.09 | 0.33±0.47 | - | - |
| 9,13-diMeC22 | - | - | - | - | - | - | 0.04±0.03 | 0.09±0.15 | - | - | - | - |
| z-C23-1 | - | - | - | - | - | - | - | - | - | - | - | - |
| z-C23-2 | - | - | - | - | - | - | - | - | 0.04±0.03 | 0.20±0.44 | - | - |
| z-C23-3 | - | - | - | - | - | - | - | - | - | - | - | - |
| z-C23-4 | - | - | - | - | 0.49±0.60 | 0.38±0.74 | - | - | - | - | - | - |
| n-C23 | - | - | 0.18±0.06 | 0.15±0.08 | 0.28±0.13 | 0.38±0.39 | 0.18±0.11 | 0.16±0.12 | 0.32±0.17 | 1.70±1.33 | 0.19±0.18 | 0.96±1.43 |
| 13-MeC23 | - | - | - | - | - | - | 0.24±0.03 | 0.27±0.23 | - | - | - | - |
| 11-;9-MeC23 | - | - | - | - | - | - | 0.18±0.02 | 0.34±0.47 | - | - | - | - |
| 5-MeC23 | - | - | 0.05±0.02 | 0.12±0.04 | - | - | - | - | - | - | - | - |
| n-C24 | 1.56±1.39 | 1.12±1.30 | 0.16±0.07 | 0.13±0.06 | 1.68±0.66 | 0.81±0.44 | 0.58±0.56 | 0.79±0.76 | 0.19±0.23 | 0.73±1.10 | - | 0.02±0.02 |
| 14-;12-MeC24 | - | - | - | - | - | - | 0.25±0.17 | 0.47±0.49 | - | - | - | - |
| 13-MeC24 | 0.44±0.43 | 0.30±0.86 | - | - | - | - | - | - | - | - | - | - |
| 9-MeC24 | - | - | - | - | 0.22±0.11 | 0.15±0.11 | - | - | - | - | - | - |
| 11-MeC24 | 0.62±0.75 | 0.39±1.10 | - | - | 0.24±0.06 | 0.13±0.06 | - | - | - | - | - | - |
| 18-;6-MeC24 | - | - | - | - | - | - | 0.12±0.12 | 0.29±0.63 | - | - | - | - |
| 13,15-diMeC24 | - | - | - | - | - | - | 0.80±0.81 | 0.49±0.64 | - | - | - | - |
| 11,15-diMeC24 | 0.45±0.44 | 0.38±0.80 | - | - | - | - | - | - | - | - | - | - |
| 11,13-diMeC24 | 0.49±0.66 | 0.24±0.44 | - | - | - | - | - | - | - | - | - | - |
| 9,13-diMeC24 | - | - | - | - | - | - | 1.59±1.59 | 0.56±0.58 | - | - | - | - |
| z-C25-1 | - | - | - | - | - | - | - | - | - | - | - | - |
| z-C25-2 | - | - | - | - | - | - | - | - | - | - | - | - |
| z-C25-3 | - | - | - | - | - | - | - | - | - | - | - | - |
| z-C25-4 | - | - | - | - | - | - | - | - | 16.92±5.74 | 5.60±4.01 | - | - |
| z-C25-5 | - | - | - | - | - | - | - | - | 0.22±0.11 | 0.27±0.13 | - | - |
| z-C25-6 | - | - | - | - | - | - | - | - | - | - | - | - |
| z-C25-7 | - | - | - | - | 0.44±0.55 | 0.11±0.07 | - | - | - | - | - | - |
| n-C25 | 20.99±11.58 | 25.52±8.48 | 0.27±0.04 | 1.20±0.43 | 0.34±0.08 | 0.90±1.17 | 3.64±1.58 | 2.10±2.61 | 13.11±3.43 | 14.32±6.05 | - | 0.19±0.30 |
| 13-;11-;9-MeC25 | - | - | - | - | - | - | - | - | - | - | - | - |
| 13-;11-MeC25 | 0.74±0.36 | 1.73±0.89 | - | - | 0.26±0.21 | 0.19±0.16 | 0.08±0.08 | 0.11±0.17 | - | - | - | - |
| 9-MeC25 | - | - | 0.77±0.17 | 2.93±0.57 | - | - | - | - | - | - | - | - |
| 7-MeC25 | - | - | 0.09±0.02 | 0.54±0.14 | - | - | - | - | - | - | - | - |
| 5-MeC25 | - | - | 1.15±0.14 | 2.69±0.46 | - | - | - | - | - | - | - | - |
| 4-MeC25 | - | - | 1.20±0.16 | 2.33±0.39 | - | - | - | - | - | - | - | - |
| 3-MeC25 | - | - | - | - | - | - | - | - | - | - | - | - |
| n-C26 | 1.32±0.55 | 1.73±0.38 | 0.44±0.06 | 0.77±0.27 | 1.39±0.25 | 1.13±0.54 | 1.09±0.27 | 1.11±0.33 | 0.35±0.26 | 1.20±0.76 | - | - |
| 14-;13-MeC26 | 0.50±0.23 | 0.88±0.78 | - | - | - | - | - | - | - | - | - | - |
| 13-MeC26 | - | - | - | - | - | - | 0.18±0.05 | 0.26±0.15 | - | - | - | - |
| 11-MeC26 | 0.22±0.14 | 0.35±0.96 | - | - | - | - | - | - | - | - | - | - |
| 10-MeC26 | - | - | 0.15±0.02 | 0.32±0.12 | - | - | - | - | - | - | - | - |
| 8-MeC26 | - | - | - | - | - | - | - | - | - | - | - | - |
| 6-MeC26 | - | - | 1.05±0.25 | 1.40±0.32 | - | - | - | - | - | - | - | - |
| 5-MeC26 | - | - | - | - | 0.14±0.16 | 0.13±0.10 | - | - | - | - | - | - |
| 13,15-; 11,15-diMeC26 | - | - | - | - | - | - | - | - | - | - | - | - |
| 11,13-; 9,13-diMeC26 | - | - | - | - | - | - | 0.11±0.03 | 0.14±0.11 | - | - | - | - |
| 4-MeC26 | - | - | - | - | - | - | - | - | - | - | - | - |
| x-MeC26 | - | - | - | - | - | - | - | - | - | - | - | - |
| z-C27-1 | - | - | - | - | - | - | - | - | - | - | - | - |
| z-C27-2 | - | - | - | - | - | - | - | - | - | - | - | - |
| z-C27-3 | - | - | - | - | - | - | - | - | - | - | - | - |
| z-C27-4 | - | - | - | - | - | - | - | - | - | - | - | - |
| z-C27-5 | - | - | - | - | - | - | - | - | 2.87±1.05 | 4.34±1.55 | - | - |
| z-C27-6 | - | - | - | - | - | - | - | - | - | - | - | - |
| z-C27-7 | - | - | - | - | - | - | - | - | - | - | - | - |
| n-C27 | 14.62±2.91 | 15.72±6.69 | 0.21±0.09 | 0.19±0.07 | 6.59±4.96 | 3.45±1.67 | 44.29±2.52 | 38.95±2.95 | 4.21±0.46 | 7.26±1.63 | 0.05±0.01 | 0.24±0.44 |
| alkadiene-C28-1 | - | - | - | - | - | - | - | - | - | - | - | - |
| 15-;13-;11-;9-MeC27 | - | - | 12.84±1.39 | 19.76±8.26 | - | - | - | - | - | - | - | - |
| 15-;13-MeC27 | 4.72±2.14 | 11.47±5.11 | - | - | 0.77±1.08 | 0.22±0.22 | - | - | - | - | - | - |
| 13-;11-;9-MeC27 | - | - | - | - | - | - | - | - | - | - | - | - |
| 13-;11-MeC27 | - | - | - | - | - | - | 0.77±1.23 | 0.15±0.10 | - | - | - | - |
| 11-;9-MeC27 | 2.21±1.18 | 3.49±2.08 | - | - | - | - | - | - | - | - | - | - |
| 13-MeC27 | - | - | - | - | - | - | - | - | - | - | - | - |
| 9-MeC27 | - | - | - | - | 0.31±0.34 | 0.06±0.04 | 0.43±0.60 | 0.14±0.14 | - | - | - | 0.07±0.11 |
| 7-MeC27 | - | - | 29.64±1.25 | 23.56±3.82 | - | - | - | - | - | - | - | - |
| 11,13-diMeC27 | - | - | - | - | - | - | 1.08±1.40 | 0.25±0.16 | - | - | - | - |
| 5-MeC27 | - | - | 3.14±0.20 | 3.67±0.72 | - | - | - | - | - | - | - | - |
| 3-MeC27 | - | - | 0.76±0.07 | 1.06±0.30 | - | - | - | - | - | - | - | - |
| 5,11-diMeC27 | - | - | 4.18±1.05 | 6.74±1.42 | - | - | - | - | - | - | - | - |
| x,y-diMeC27-1 | - | - | - | - | - | - | - | - | - | - | - | - |
| x,y-diMeC27-2 | - | - | - | - | - | - | - | - | - | - | - | - |
| z-C28-1 | - | - | - | - | - | - | - | - | - | - | - | - |
| n-C28 | 0.91±0.32 | 0.77±0.52 | 1.14±0.10 | 2.39±2.18 | 2.92±0.28 | 2.05±0.98 | 2.93±0.33 | 3.54±1.88 | 0.33±0.36 | 1.13±0.89 | 0.07±0.02 | 0.13±0.05 |
| 15-;13-;11-MeC28 | - | - | - | - | - | - | 0.09±0.03 | 0.51±0.99 | - | - | - | - |
| 14-;13-;12-;11-MeC28 | - | - | - | - | - | - | - | - | - | - | - | - |
| 14-;13-;12-MeC28 | - | - | - | - | - | - | - | - | - | - | - | - |
| 14-MeC28 | 0.66±0.38 | 1.46±0.93 | - | - | - | - | - | - | - | - | - | - |
| 13-MeC28 | 0.43±0.24 | 0.59±0.55 | 1.66±0.26 | 1.14±0.53 | - | - | - | - | - | - | - | - |
| 12-;10-;8-;4-MeC28 | - | - | 11.46±0.81 | 9.31±1.98 | - | - | - | - | - | - | - | - |
| 5-MeC28 | - | - | - | - | - | - | 0.07±0.02 | 0.17±0.16 | - | - | - | - |
| 4-MeC28 | - | - | - | - | - | - | - | - | - | - | - | - |
| 3-MeC28 | - | - | 4.00±0.50 | 2.38±1.04 | - | - | - | - | - | - | - | - |
| 9,11-diMeC28 | - | - | - | - | - | - | 0.11±0.12 | 0.09±0.09 | - | - | - | - |
| 8,12-diMeC28 | - | - | - | - | - | - | - | - | - | - | - | - |
| 5,11-diMeC28 | - | - | - | - | - | - | 0.05±0.04 | 0.10±0.09 | - | - | - | - |
| x,y-diMeC28 | - | - | - | - | - | - | - | - | - | - | - | - |
| alkadiene-C29-1 | - | - | - | - | - | - | - | - | - | - | - | - |
| z-C29-1 | - | - | - | - | - | - | - | - | - | - | - | - |
| z-C29-2 | - | - | - | - | - | - | - | - | - | - | - | - |
| z-C29-3 | - | - | - | - | - | - | - | - | - | - | - | - |
| z-C29-4 | - | - | 0.28±0.07 | 0.30±0.10 | - | - | - | - | - | - | - | - |
| z-C29-5 | - | - | - | - | - | - | - | - | - | - | - | - |
| z-C29-6 | - | - | - | - | - | - | - | - | - | - | - | - |
| z-C29-7 | - | - | - | - | - | - | - | - | - | - | - | - |
| z-C29-8 | - | - | - | - | - | - | - | - | 3.20±1.09 | 2.85±0.95 | - | - |
| z-C29-9 | - | - | - | - | - | - | - | - | - | - | - | - |
| z-C29-10 | - | - | - | - | - | - | - | - | - | - | - | - |
| n-C29 | 28.10±13.20 | 13.32±8.26 | 0.11±0.03 | 0.10±0.04 | 44.24±7.09 | 44.08±6.98 | 32.61±3.53 | 36.94±5.73 | 9.20±1.44 | 8.03±1.50 | 5.78±0.61 | 6.72±2.47 |
| unknown | - | - | - | - | - | - | - | - | - | - | - | - |
| unknown | - | - | - | - | - | - | - | - | - | - | - | - |
| 15-;13-;11-;9-MeC29 | - | - | - | - | - | - | - | - | - | - | - | - |
| 15-;13-;11-MeC29 | 5.73±3.03 | 11.94±6.23 | - | - | - | - | 1.45±1.41 | 1.21±1.72 | - | - | 0.35±0.08 | 1.23±1.62 |
| 13-MeC29 | - | - | - | - | 0.50±0.64 | 0.12±0.11 | - | - | - | - | - | - |
| 11-MeC29 | - | - | - | - | 0.27±0.35 | 0.06±0.04 | - | - | - | - | - | - |
| 9-MeC29 | - | - | - | - | - | - | - | - | - | - | - | - |
| 7-MeC29 | - | - | - | - | - | - | - | - | - | - | - | 0.03±0.03 |
| 13,x-diMeC29 | - | - | - | - | - | - | - | - | - | - | - | 0.05±0.05 |
| 11,13-diMeC29 | - | - | 7.38±2.03 | 5.65±2.27 | - | - | - | - | - | - | - | - |
| 5-MeC29 | - | - | 6.31±0.27 | 4.65±0.97 | - | - | - | - | - | - | - | - |
| 4-MeC29 | - | - | - | - | - | - | - | - | - | - | - | - |
| 3-MeC29 | - | - | 1.36±0.17 | 0.82±0.26 | 0.09±0.04 | 0.07±0.07 | - | - | - | - | - | - |
| 15,17-diMeC29 | 2.40±1.38 | 4.65±3.14 | - | - | - | - | - | - | - | - | - | - |
| 13,17-; 11,15-diMeC29 | - | - | - | - | - | - | - | - | - | - | - | - |
| 9,15-; 9,13-diMeC29 | - | - | - | - | - | - | - | - | - | - | - | - |
| 9,15-diMeC29 | - | - | - | - | - | - | - | - | - | - | - | - |
| 5,11-diMeC29 | - | - | 3.08±0.40 | 1.79±0.27 | - | - | - | - | - | - | - | - |
| 3,13-diMeC29 | - | - | - | - | - | - | - | - | - | - | - | - |
| 3,11-diMeC29 | - | - | 1.69±0.19 | 1.12±0.33 | - | - | - | - | - | - | - | - |
| x,y-diMeC29-1 | - | - | - | - | - | - | - | - | - | - | - | - |
| x,y-diMeC29-2 | - | - | - | - | - | - | - | - | - | - | - | - |
| z-C30-1 | - | - | - | - | - | - | - | - | 0.33±0.09 | 0.66±0.23 | - | - |
| n-C30 | 1.69±0.68 | 0.78±0.61 | 3.70±0.34 | 1.81±0.56 | 3.95±0.49 | 3.14±1.15 | 1.54±1.44 | 3.20±4.73 | 0.86±0.35 | 2.11±0.85 | 0.11±0.03 | 0.35±0.14 |
| 15-;14-;13-;12-;11-;10-MeC30 | - | - | - | - | - | - | - | - | - | - | - | - |
| 15-;14-;13-MeC30 | - | - | - | - | - | - | - | - | - | - | - | 0.13±0.18 |
| 13-MeC30 | - | - | - | - | - | - | - | - | - | - | - | - |
| 12-;11-MeC30 | - | - | - | - | - | - | 0.12±0.07 | 0.36±0.20 | - | - | - | - |
| 13,15-; 12,14-MeC30 | - | - | - | - | - | - | - | - | - | - | - | - |
| 5,13-MeC30 | - | - | - | - | - | - | - | - | - | - | - | - |
| 5,11-MeC30 | - | - | - | - | - | - | - | - | - | - | - | - |
| 4-MeC30 | - | - | - | - | - | - | - | - | - | - | - | - |
| x,y-diMeC30-1 | - | - | - | - | - | - | - | - | - | - | - | - |
| z-C31-1 | - | - | - | - | - | - | - | - | - | - | - | - |
| z-C31-2 | - | - | - | - | - | - | - | - | - | - | - | - |
| z-C31-3 | - | - | - | - | - | - | - | - | - | - | - | - |
| z-C31-4 | - | - | - | - | - | - | - | - | - | - | - | - |
| z-C31-5 | - | - | - | - | - | - | - | - | - | - | - | - |
| z-C31-8 | - | - | - | - | - | - | - | - | 23.97±2.78 | 16.59±3.56 | - | - |
| z-C31-9 | - | - | - | - | - | - | - | - | - | - | - | - |
| n-C31 | 11.20±4.68 | 3.18±2.31 | - | - | 30.34±3.98 | 38.66±7.29 | 3.97±1.47 | 6.00±1.30 | 20.71±6.10 | 19.22±5.66 | 2.16±0.46 | 5.80±2.24 |
| 15-; 13-; 11-;9-;7-MeC31 | - | - | - | - | - | - | - | - | - | - | - | - |
| 15-; 13-; 11-;9-MeC31 | - | - | - | - | - | - | - | - | - | - | 0.60±0.10 | 1.93±2.90 |
| 15-; 13-; 11-MeC31 | - | - | - | - | - | - | - | - | - | - | - | - |
| 15-; 13-MeC31 | - | - | - | - | - | - | - | - | - | - | - | - |
| 7-MeC31 | - | - | - | - | - | - | - | - | - | - | - | 0.03±0.02 |
| 13,17-diMeC31 | - | - | - | - | - | - | - | - | - | - | 0.21±0.03 | 0.27±0.31 |
| 13,17-; 11,15-diMeC31 | - | - | - | - | - | - | - | - | - | - | - | - |
| 13,17-; 13,15-; 11,15-diMeC31 | - | - | - | - | - | - | - | - | - | - | - | - |
| 9,13-diMeC31 | - | - | - | - | - | - | - | - | - | - | - | - |
| x,y-diMeC31 | - | - | - | - | - | - | - | - | - | - | - | - |
| 3-MeC31 | - | - | - | - | - | - | - | - | - | - | - | - |
| z-C32-1 | - | - | - | - | - | - | - | - | 0.17±0.05 | 0.54±0.21 | - | - |
| n-C32 | - | - | - | - | 1.71±0.97 | 1.13±0.90 | - | - | 0.51±0.23 | 1.69±0.75 | - | 0.07±0.07 |
| 16-;15-;14-;13-MeC32 | - | - | - | - | - | - | - | - | - | - | - | - |
| 15-;14-;13-;12-;11-MeC32 | - | - | - | - | - | - | - | - | - | - | - | - |
| 14-; 12-MeC32 | - | - | - | - | - | - | - | - | - | - | - | - |
| 13-;12-;11-MeC32 | - | - | - | - | - | - | - | - | - | - | - | 0.28±0.30 |
| 13-MeC32 | - | - | - | - | - | - | - | - | - | - | - | - |
| 4-MeC32 | - | - | - | - | - | - | - | - | - | - | - | - |
| z-C33-1 | - | - | - | - | - | - | - | - | - | - | - | - |
| z-C33-2 | - | - | - | - | - | - | - | - | 0.76±0.27 | 5.17±1.83 | - | - |
| n-C33 | - | - | - | - | 1.09±0.47 | 1.39±0.88 | - | - | 1.60±0.50 | 5.96±2.17 | 0.07±0.02 | 0.58±0.68 |
| 17-;15-;13-MeC33 | - | - | - | - | - | - | - | - | - | - | - | - |
| 15-;13-;11-;9-MeC33 | - | - | - | - | - | - | - | - | - | - | 2.57±0.23 | 3.22±3.47 |
| 15-;13-;11-MeC33 | - | - | - | - | - | - | - | - | - | - | - | - |
| 15-; 13-MeC33 | - | - | - | - | - | - | - | - | - | - | - | - |
| 13,17-; 11,15-diMeC33 | - | - | - | - | - | - | - | - | - | - | - | - |
| 13,17-diMeC33 | - | - | - | - | - | - | - | - | - | - | 6.59±0.59 | 3.95±0.99 |
| 13,15-; 11,13-diMeC33 | - | - | - | - | - | - | - | - | - | - | - | - |
| 7,X-diMeC33 | - | - | - | - | - | - | - | - | - | - | 0.29±0.07 | 0.15±0.11 |
| 3,15-; 3,13-diMeC33 | - | - | - | - | - | - | - | - | - | - | 0.32±0.09 | - |
| n-C34 | - | - | - | - | - | - | - | - | - | - | - | 0.17±0.06 |
| 17-;16-;14-;11-MeC34 | - | - | - | - | - | - | - | - | - | - | - | - |
| 17-;15-;13-MeC34 | - | - | - | - | - | - | - | - | - | - | - | - |
| 15-;14-;13-;12-MeC34 | - | - | - | - | - | - | - | - | - | - | 1.35±0.20 | 1.06±0.34 |
| 12,16-diMeC34 | - | - | - | - | - | - | - | - | - | - | 2.41±0.34 | 1.45±0.41 |
| 8,16-; 8,12-diMeC34 | - | - | - | - | - | - | - | - | - | - | 0.08±0.04 | - |
| x,y-diMeC34 - 1 | - | - | - | - | - | - | - | - | - | - | 0.09±0.02 | - |
| x,y-diMeC34 - 2 | - | - | - | - | - | - | - | - | - | - | 0.10±0.01 | - |
| n-C35 | - | - | - | - | - | - | - | - | - | - | 0.37±0.06 | 0.82±0.20 |
| 17-,15-,13-,11-,9-;7-MeC35 | - | - | - | - | - | - | - | - | - | - | 19.86±0.51 | 15.37±1.85 |
| 17-;15-;13-;11-MeC35 | - | - | - | - | - | - | - | - | - | - | - | - |
| 17-; 15-; 13-MeC35 | - | - | - | - | - | - | - | - | - | - | - | - |
| 13-MeC35 | - | - | - | - | - | - | - | - | - | - | - | - |
| 15,19-; 13,17-diMeC35 | - | - | - | - | - | - | - | - | - | - | 23.91±0.82 | 18.62±3.71 |
| 13,19-; 13,17-diMeC35 | - | - | - | - | - | - | - | - | - | - | - | - |
| 13,17-diMeC35 | - | - | - | - | - | - | - | - | - | - | - | - |
| 11,19-diMeC35 | - | - | - | - | - | - | - | - | - | - | - | - |
| 9,19-; 9,17-diMeC35 | - | - | - | - | - | - | - | - | - | - | - | - |
| 7,21-; 7,19-diMeC35 | - | - | - | - | - | - | - | - | - | - | - | - |
| 7,15-; 7,13-diMeC35 | - | - | - | - | - | - | - | - | - | - | - | 1.08±0.52 |
| 7,11-diMeC35 | - | - | - | - | - | - | - | - | - | - | 1.60±0.15 | - |
| 5,19-; 5,17-diMeC35 | - | - | - | - | - | - | - | - | - | - | - | - |
| 5,15-diMeC35 | - | - | - | - | - | - | - | - | - | - | 3.24±0.11 | 3.02±0.92 |
| 3,17-; 3,13-diMeC35 | - | - | - | - | - | - | - | - | - | - | - | 1.00±0.26 |
| 3,15-diMeC35 | - | - | - | - | - | - | - | - | - | - | 1.23±0.09 | - |
| 3,11-; 3,9-diMeC35 | - | - | - | - | - | - | - | - | - | - | 2.78±0.10 | 2.29±0.35 |
| 17-;15-;13-MeC36 | - | - | - | - | - | - | - | - | - | - | 2.22±0.50 | 2.07±0.72 |
| 10,16-; 10,14-diMeC36 | - | - | - | - | - | - | - | - | - | - | - | - |
| 8,16-; 8,12-diMeC36 | - | - | - | - | - | - | - | - | - | - | 0.31±0.36 | 0.33±0.44 |
| 3,17-; 3,11-diMeC36 | - | - | - | - | - | - | - | - | - | - | 0.25±0.06 | - |
| n-C37 | - | - | - | - | - | - | - | - | - | - | 0.24±0.05 | 0.69±0.29 |
| 17-;15-;13-MeC37 | - | - | - | - | - | - | - | - | - | - | - | - |
| 15-;13-;11-;9-;7-MeC37 | - | - | - | - | - | - | - | - | - | - | 8.92±0.49 | 10.17±1.36 |
| 15,19-; 13,19-MeC37 | - | - | - | - | - | - | - | - | - | - | - | - |
| 13,21-; 13,19-; 11,15-diMeC37 | - | - | - | - | - | - | - | - | - | - | 8.88±0.56 | 10.90±2.84 |
| 11,21-; 11,19-diMeC37 | - | - | - | - | - | - | - | - | - | - | - | - |
| 7,17-; 7,15-; 7,13-diMeC37 | - | - | - | - | - | - | - | - | - | - | 0.72±0.05 | 0.74±0.19 |
| 5,19-; 5,17-diMeC37 | - | - | - | - | - | - | - | - | - | - | - | - |
| x,y-diMeC37 | - | - | - | - | - | - | - | - | - | - | - | 1.59±0.75 |
| 3,x-diMeC37 | - | - | - | - | - | - | - | - | - | - | 1.15±0.08 | - |
| x,y,z-triMeC37-1 | - | - | - | - | - | - | - | - | - | - | - | - |
| x,y,z-triMeC37-2 | - | - | - | - | - | - | - | - | - | - | - | - |
| x,y,z-triMeC37-3 | - | - | - | - | - | - | - | - | - | - | - | - |
| x,y,z-triMeC37-4 | - | - | - | - | - | - | - | - | - | - | - | - |
| 17-; 13-; 11-MeC38 | - | - | - | - | - | - | - | - | - | - | - | - |
| 14-;12-MeC38 | - | - | - | - | - | - | - | - | - | - | 0.28±0.03 | 0.49±0.16 |
| n-C39 | - | - | - | - | - | - | - | - | - | - | - | - |
| 17-;15-MeC39 | - | - | - | - | - | - | - | - | - | - | - | - |
| 15-;13-;11-MeC39 | - | - | - | - | - | - | - | - | - | - | 0.43±0.13 | 1.01±0 |
| 13,15-; 11,13-diMeC39 | - | - | - | - | - | - | - | - | - | - | - | - |
| 11,21-; 11,19-diMeC39 | - | - | - | - | - | - | - | - | - | - | - | - |
| 13,X-diMeC39 | - | - | - | - | - | - | - | - | - | - | - | 0.67±0.43 |
| 11,x-diMeC39 | - | - | - | - | - | - | - | - | - | - | 0.21±0.06 | - |
| x,y-diMeC39-1 | - | - | - | - | - | - | - | - | - | - | - | - |
| 5,19-; 5,17-diMeC39 | - | - | - | - | - | - | - | - | - | - | - | - |
| x,y,z-triMeC39-1 | - | - | - | - | - | - | - | - | - | - | - | - |
| x,y,z-triMeC39-2 | - | - | - | - | - | - | - | - | - | - | - | - |
| 13-MeC40 | - | - | - | - | - | - | - | - | - | - | - | - |
| 11-MeC40 | - | - | - | - | - | - | - | - | - | - | - | - |

Cont Table S1. Groups II and III: Relative contribution (%) of cuticular hydrocarbon composition in Epiponini wasps from the present study. In parenthesis is presented the number of individuals analyzed per group from each species. sd = standard deviation. Considering that we did not perform the derivatization of the chemical samples, we could not confirm the right position of the double bounds for the alkenes identified. Thus, each of them was represented as unique in the table.

| caste syndromes | (II) Reproductive females isometrically different from workers | | | | | | (III) Reproductive females allometrically different from workers | | | |
| --- | --- | --- | --- | --- | --- | --- | --- | --- | --- | --- |
| species | *Brachygastra augusti* | | *Charterginus* sp. | | *Polybia paulista* | | *Apoica flavissima* | | *Agelaia pallipes* | |
| Compounds | Queens (11) | Workers (15) | Queens (6) | Workers (25) | Queens (16) | Workers (16) | Queens (7) | Workers (8) | Queens (12) | Workers (22) |
|  | mean±sd | mean±sd | mean±sd | mean±sd | mean±sd | mean±sd | mean±sd | mean±sd | mean±sd | mean±sd |
| n-C18 | - | - | - | - | - | - | - | - | - | - |
| n-C19 | - | - | - | - | - | - | - | - | - | - |
| n-C20 | 1.28±1.14 | 1.02±0.58 | - | - | - | - | - | - | - | - |
| n-C21 | 0.27±0.27 | 0.09±0.18 | - | - | - | - | - | - | 0.05±0.01 | 0.04±0.04 |
| n-C22 | 2.29±2.30 | 1.13±0.64 | - | - | - | 0.12±0.27 | - | - | 0.35±0.18 | 0.28±0.18 |
| 9,13-diMeC22 | - | - | - | - | - | - | - | - | - | - |
| z-C23-1 | - | - | - | - | 0.48±0.43 | - | - | - | - | - |
| z-C23-2 | - | - | - | - | - | - | - | - | - | - |
| z-C23-3 | - | - | - | 0.27±0.46 | - | - | - | - | - | - |
| z-C23-4 | - | - | - | - | - | - | - | - | - | - |
| n-C23 | 3.12±2.87 | 0.77±1.29 | 1.18±0.30 | 2.49±3.16 | 0.22±0.18 | 2.89±6.23 | 0.07±0.06 | 0.03±0.01 | 0.65±0.25 | - |
| 13-MeC23 | - | - | - | - | - | - | - | - | - | - |
| 11-;9-MeC23 | - | - | - | - | - | - | - | - | - | - |
| 5-MeC23 | - | - | - | - | - | - | - | - | - | - |
| n-C24 | 3.28±3.01 | 0.86±1.32 | 0.17±0.05 | 0.15±0.08 | 0.09±0.07 | 0.16±0.12 | 0.14±0.11 | 0.12±0.07 | 0.45±0.12 | 0.35±0.23 |
| 14-;12-MeC24 | - | - | - | - | - | - | - | - | - | - |
| 13-MeC24 | - | - | - | - | - | - | - | - | - | - |
| 9-MeC24 | - | - | - | - | - | - | - | - | - | - |
| 11-MeC24 | - | - | - | - | - | - | - | - | - | - |
| 18-;6-MeC24 | 1.52±3.14 | 0.17±0.34 | - | - | - | - | - | - | - | - |
| 13,15-diMeC24 | - | - | - | - | - | - | - | - | - | - |
| 11,15-diMeC24 | - | - | - | - | - | - | - | - | - | - |
| 11,13-diMeC24 | - | - | - | - | - | - | - | - | - | - |
| 9,13-diMeC24 | - | - | - | - | - | - | - | - | - | - |
| z-C25-1 | 0.98±1.06 | 0.34±0.80 | - | - | - | - | - | - | - | - |
| z-C25-2 | - | - | - | - | 0.17±0.13 | 0.23±0.23 | - | - | - | - |
| z-C25-3 | - | - | - | - | 0.27±0.74 | 0.36±0.85 | - | - | - | - |
| z-C25-4 | - | - | - | - | - | - | - | - | - | - |
| z-C25-5 | - | - | - | - | - | - | - | - | - | - |
| z-C25-6 | - | - | 0.28±0.06 | 1.26±0.97 | - | - | - | - | - | - |
| z-C25-7 | - | - | - | - | - | - | - | - | - | - |
| n-C25 | 4.64±2.39 | 4.37±1.27 | 4.46±0.55 | 2.87±1.37 | 1.88±1.66 | 4.63±2.36 | 0.93±0.26 | 1.16±0.35 | 0.75±0.18 | 0.62±0.34 |
| 13-;11-;9-MeC25 | - | - | - | - | - | - | - | - | 0.29±0.03 | 0.26±0.10 |
| 13-;11-MeC25 | 0.68±0.74 | 0.81±0.39 | - | - | - | 1.00±0.75 | - | - | - | - |
| 9-MeC25 | - | - | - | - | - | - | - | - | - | - |
| 7-MeC25 | - | - | 0.02±0.01 | 0.14±0.08 | - | - | - | - | - | - |
| 5-MeC25 | - | - | 0.04±0.01 | 0.17±0.08 | - | 0.23±0.28 | - | - | - | - |
| 4-MeC25 | - | - | - | - | - | - | - | - | - | - |
| 3-MeC25 | - | - | 0.08±0.01 | 0.56±0.31 | 0.22±0.43 | 0.65±0.61 | - | - | 0.14±0.02 | 0.10±0.04 |
| n-C26 | 1.86±1.52 | 1.01±0.85 | 0.86±0.17 | 0.73±0.16 | 0.56±0.22 | 1.14±1.44 | 0.31±0.30 | 0.30±0.16 | 0.45±0.09 | 0.36±0.14 |
| 14-;13-MeC26 | - | - | - | - | - | - | - |  | - | - |
| 13-MeC26 | 0.30±0.38 | 0.04±0.05 | - | - | - | - | - | - | - | - |
| 11-MeC26 | - | - | - | - | - | - | - | - | - | - |
| 10-MeC26 | - | - | - | - | - | - | - | - | - | - |
| 8-MeC26 | 0.21±0.24 | 0.08±0.15 | - | - | - | - | - | - | - | - |
| 6-MeC26 | - | - | - | - | - | - | - | - | - | - |
| 5-MeC26 | 0.07±0.08 | 0.02±0.02 | - | - | - | - | - | - | - | - |
| 13,15-; 11,15-diMeC26 | 0.33±0.41 | 0.17±0.17 | - | - | - | - | - | - | - | - |
| 11,13-; 9,13-diMeC26 | - | - | - | - | - | - | - | - | - | - |
| 4-MeC26 | 0.38±0.49 | 0.07±0.18 | - | - | - | - | - | - | - | - |
| x-MeC26 | - | - | - | - | - | - | - | - | 0.61±0.10 | 0.54±0.25 |
| z-C27-1 | 0.52±0.57 | 0.18±0.53 | - | - | - | - | - | - | - | - |
| z-C27-2 | 0.35±0.48 | 0.10±0.25 | - | - | - | - | - | - | - | - |
| z-C27-3 | - | - | - | - | 0.77±2.46 | 1.12±2.67 | - | - | - | - |
| z-C27-4 | - | - | - | - | - | - | - | - | - | 0.18±0.08 |
| z-C27-5 | - | - | - | - | - | - | - | - | - | - |
| z-C27-6 | - | - | 1.17±0.23 | 2.21±1.09 | - | - | - | - | - | - |
| z-C27-7 | - | - | 0.06±0.02 | 0.57±0.40 | - | - | - | - | - | - |
| n-C27 | 16.25±8.99 | 8.27±4.19 | 15.20±2.02 | 18.39±4.52 | 16.99±2.49 | 16.17±6.64 | 10.48±3.98 | 17.14±9.12 | 1.37±0.24 | 1.34±0.91 |
| alkadiene-C28-1 | - | - | 0.04±0.03 | 0.18±0.14 | - | - | - | - | - | - |
| 15-;13-;11-;9-MeC27 | - | - | - | - | - | - | - | - | - | - |
| 15-;13-MeC27 | - | - | - | - | - | - | - | - | - | - |
| 13-;11-;9-MeC27 | - | - | - | - | - | - | - | - | 0.48±0.07 | 0.51±0.62 |
| 13-;11-MeC27 | 1.73±2.89 | 2.59±0.75 | 0.19±0.04 | 1.75±1.05 | 6.43±2.58 | 9.37±3.45 | 0.32±0.24 | 0.35±0.44 | - | - |
| 11-;9-MeC27 | - | - | - | - | - | - | - | - | - | - |
| 13-MeC27 | - | - | - | - | - | - | 0.06±0.12 | - | - | - |
| 9-MeC27 | - | - | 0.41±0.07 | 3.86±1.69 | - | - | 0.20±0.20 | 0.15±0.09 | - | - |
| 7-MeC27 | - | - | 0.14±0.02 | 1.23±0.57 | 0.39±0.35 | 0.59±0.44 | 0.11±0.08 | 0.14±0.12 | 0.10±0.01 | 0.06±0.05 |
| 11,13-diMeC27 | - | - | - | - | 1.72±0.61 | 2.28±0.87 | - | - | - | - |
| 5-MeC27 | - | - | 0.99±0.13 | 2.30±0.62 | 0.47±0.35 | 0.70±0.41 | 0.09±0.06 | 0.08±0.09 | - | - |
| 3-MeC27 | 0.47±0.67 | 0.23±0.15 | 1.67±0.17 | 7.81±1.51 | 5.04±1.76 | 5.16±2.17 | 0.72±0.16 | 0.59±0.23 | 0.64±0.11 | 0.36±0.38 |
| 5,11-diMeC27 | - | - | - | - | - | - | - | - | - | - |
| x,y-diMeC27-1 | - | - | - | - | 0.13±0.18 | 0.27±0.26 | - | - | - | - |
| x,y-diMeC27-2 | - | - | - | - | - | - | - | - | - | 0.20±0.07 |
| z-C28-1 | 0.61±1.23 | 0.10±0.11 | - | - | - | - | - | - | - | - |
| n-C28 | 2.39±1.01 | 1.25±0.58 | 0.85±0.17 | 1.23±0.32 | 1.58±0.44 | 1.11±0.34 | 0.63±0.25 | 0.88±0.34 | 2.08±0.29 | 1.23±0.31 |
| 15-;13-;11-MeC28 | - | - | - | - | - | - | - | - | - | - |
| 14-;13-;12-;11-MeC28 | - | - | - | - | 2.10±0.50 | 2.23±0.60 | - | - | - | - |
| 14-;13-;12-MeC28 | - | - | - | - | - | - | - | - | 0.35±0.07 | 0.32±0.19 |
| 14-MeC28 | - | - | - | - | - | - | - | - | - | - |
| 13-MeC28 | - | - | - | - | - | - | - | - | - | - |
| 12-;10-;8-;4-MeC28 | - | - | - | - | - | - | - | - | - | - |
| 5-MeC28 | - | - | 0.13±0.02 | 0.25±0.09 | - | - | - | - | - | - |
| 4-MeC28 | - | - | - | - | 0.11±0.20 | 0.11±0.12 | 0.24±0.22 | 0.09±0.04 | 0.58±0.13 | - |
| 3-MeC28 | - | - | - | 0.14±0.07 | - | - | - | - | - | - |
| 9,11-diMeC28 | - | - | - | - | - | - | - | - | - | - |
| 8,12-diMeC28 | 0.18±0.16 | 0.49±0.25 | - | - | - | - | - | - | - | - |
| 5,11-diMeC28 | - | - | - | - | - | - | - | - | - | - |
| x,y-diMeC28 | - | - | - | - | - | - | - | - | 4.42±0.54 | 6.79±2.96 |
| alkadiene-C29-1 | - | - | 0.15±0.03 | 1.49±0.42 | - | - | - | - | - | - |
| z-C29-1 | - | - | - | - | - | - | 0.18±0.13 | 0.15±0.05 | - | - |
| z-C29-2 | - | - | - | - | - | - | 0.37±0.10 | 0.53±0.38 | - | - |
| z-C29-3 | 3.22±3.88 | 1.24±3.79 | - | - | - | - | - | - | - | - |
| z-C29-4 | - | - | - | - | - | - | - | - | - | - |
| z-C29-5 | - | - | - | - | 0.37±0.87 | 0.53±0.72 | - | - | - | - |
| z-C29-6 | - | - | - | - | - | - | - | - | 2.00±0.30 | 2.33±1.17 |
| z-C29-7 | - | - | - | - | - | - | - | - | 0.59±0.10 | 0.77±0.39 |
| z-C29-8 | - | - | - | - | - | - | - | - | - | - |
| z-C29-9 | - | - | 1.19±0.19 | 8.30±1.56 | - | - | - | - | - | - |
| z-C29-10 | - | - | 2.86±0.46 | 1.17±0.28 | - | - | - | - | - | - |
| n-C29 | 27.99±14.28 | 9.43±2.58 | 3.02±0.32 | 9.77±3.19 | 20.97±7.57 | 9.59±6.49 | 4.84±1.62 | 9.56±5.64 | 8.24±0.85 | 8.19±2.60 |
| unknown | - | - | 0.43±0.10 | 2.58±0.81 | - | - | - | - | - | - |
| unknown | - | - | 0.07±0.04 | 0.30±0.14 | - | - | - | - | - | - |
| 15-;13-;11-;9-MeC29 | - | - | - | - | - | - | - | - | 6.38±0.29 | 6.69±2.21 |
| 15-;13-;11-MeC29 | 1.56±1.68 | 12.95±4.48 | 0.47±0.04 | 3.64±1.59 | 22.56±3.71 | 23.84±6.31 | 1.60±0.61 | 1.47±0.85 | - | - |
| 13-MeC29 | - | - | - | - | - | - | - | - | - | - |
| 11-MeC29 | - | - | - | - | - | - | - | - | - | - |
| 9-MeC29 | - | - | 0.23±0.06 | 1.87±0.74 | - | - | 1.08±0.21 | 0.89±0.26 | - | - |
| 7-MeC29 | - | - | 0.44±0.04 | 3.03±0.99 | - | 0.04±0.12 | 0.27±0.40 | 0.20±0.16 | 0.50±0.18 | 0.60±1.10 |
| 13,x-diMeC29 | - | - | - | - | - | - | - | - | - | - |
| 11,13-diMeC29 | - | - | - | - | - | - | - | - | - | - |
| 5-MeC29 | - | - | 0.20±0.08 | 1.07±0.33 | 0.28±0.05 | 0.31±0.16 | 0.13±0.06 | 0.19±0.26 | 0.18±0.07 | 0.14±0.09 |
| 4-MeC29 | - | - | - | - | 0.17±0.16 | - | - | - | - | - |
| 3-MeC29 | - | - | 0.52±0.07 | 3.23±0.86 | 1.31±0.54 | 1.00±0.83 | 3.56±0.58 | 2.51±1.51 | 2.26±0.25 | 1.61±0.48 |
| 15,17-diMeC29 | - | - | - | - | - | - | - | - | - | - |
| 13,17-; 11,15-diMeC29 | - | - | - | - | - | - | - | - | 0.61±0.14 | 0.56±0.35 |
| 9,15-; 9,13-diMeC29 | - | - | - | - | - | - | - | - | 0.38±0.11 | 0.35±0.37 |
| 9,15-diMeC29 | 1.60±1.35 | 0.77±0.45 | - | - | - | - | - | - | - | - |
| 5,11-diMeC29 | - | - | - | - | - | - | - | - | - | - |
| 3,13-diMeC29 | - | - | - | - | - | - | - | - | - | 0.31±0.46 |
| 3,11-diMeC29 | - | - | - | - | - | - | - | - | - | - |
| x,y-diMeC29-1 | - | - | - | - | 0.73±0.16 | 0.70±0.94 | - | - | - | - |
| x,y-diMeC29-2 | - | - | - | - | 3.65±0.44 | 2.24±1.52 | - | - | - | - |
| z-C30-1 | - | - | - | - | - | - | - | - | - | - |
| n-C30 | - | - | - | - | - | - | - | - | 3.67±0.40 | 1.77±0.50 |
| 15-;14-;13-;12-;11-;10-MeC30 | - | - | - | - | - | - | - | - | 1.49±0.08 | 1.39±0.32 |
| 15-;14-;13-MeC30 | - | - | - | - | 1.15±0.19 | 1.07±0.41 | - | - | - | - |
| 13-MeC30 | - | - | - | - | - | - | 0.31±0.07 | 0.26±0.07 | - | - |
| 12-;11-MeC30 | 0.32±0.20 | 0.07±0.13 | - | - | - | - | - | - | - | - |
| 13,15-; 12,14-MeC30 | 0.10±0.15 | 1.62±0.59 | - | - | - | - | - | - | - | - |
| 5,13-diMeC30 | - | - | - | - | - | - | - | - | 0.68±0.10 | - |
| 5,11-diMeC30 | 3.12±3.40 | 1.04±3.21 | - | - | - | - | - | - | - | - |
| 4-MeC30 | - | - | - | - | - | - | 0.23±0.07 | 0.15±0.11 | 1.46±0.18 | 0.67±0.25 |
| x,y-diMeC30-1 | - | - | - | - | - | - | - | - | - | 1.12±0.38 |
| z-C31-1 | - | - | - | - | - | - | 0.16±0.12 | 0.12±0.06 | - | - |
| z-C31-2 | - | - | - | - | - | - | 0.09±0.06 | 0.10a±0.05 | - | - |
| z-C31-3 | - | - | - | - | 0.25±0.42 | 1.75±3.20 | - | - | - | - |
| z-C31-4 | - | - | - | - | - | - | - | - | 4.85±0.40 | 5.42±1.17 |
| z-C31-5 | - | - | - | - | - | - | - | - | - | 0.44±0.17 |
| z-C31-8 | - | - | - | - | - | - | - | - | - | - |
| z-C31-9 | - | - | - | 0.18±0.24 | - | - | - | - | - | - |
| n-C31 | 10.60±11.09 | 4.06±1.37 | 0.13±0.05 | 0.61±0.16 | 2.45±0.99 | 1.41±1.46 | 0.65±0.34 | 0.92±0.53 | 3.84±0.34 | 4.42±1.29 |
| 15-; 13-; 11-;9-;7-MeC31 | - | - | - | - | - | - | - | - | 15.82±1.15 | 16.77±1.82 |
| 15-; 13-; 11-;9-MeC31 | - | - | - | - | - | - | - | - | - | - |
| 15-; 13-; 11-MeC31 | 1.32±1.65 | 19.97±7.25 | 0.05±0.01 | 0.35±0.13 | - | - | 9.29±1.19 | 7.72±2.32 | - | - |
| 15-; 13-MeC31 | - | - | - | - | 5.15±0.88 | 5.34±1.31 | - | - | - | - |
| 7-MeC31 | - | - | - | - | - | - | - | - | - | - |
| 13,17-diMeC31 | - | - | - | - | - | - | - | - | - | - |
| 13,17-; 11,15-diMeC31 | - | - | - | - | - | - | - | - | 5.14±0.45 | 4.54±1.43 |
| 13,17-; 13,15-; 11,15-diMeC31 | - | - | - | - | - | - | 1.33±0.26 | 1.00±0.42 | - | - |
| 9,13-diMeC31 | - | - | - | - | - | - | - | - | 0.92±0.24 | 1.70±1.00 |
| x,y-diMeC31 | - | - | - | - | 0.78±0.26 | 0.63±0.46 | - | - | - | - |
| 3-MeC31 | - | - | 0.03±0.01 | 0.25±0.13 | - | - | - | - | 0.17±0.10 | - |
| z-C32-1 | - | - | - | - | - | - | - | - | - | - |
| n-C32 | - | - | - | - | - | - | - | - | 0.61±0.11 | 0.44±0.28 |
| 16-;15-;14-;13-MeC32 | - | - | - | - | 0.12±0.12 | - | - | - | - | - |
| 15-;14-;13-;12-;11-MeC32 | - | - | - | - | - | - | - | - | 1.15±0.19 | 1.42±0.29 |
| 14-; 12-MeC32 | - | - | - | - | - | - | 0.62±0.23 | 0.42±0.12 | - | - |
| 13-;12-;11-MeC32 | - | - | - | - | - | - | - | - | - | - |
| 13-MeC32 | 0.13±0.17 | 1.56±0.50 | - | - | - | - | - | - | - | - |
| 4-MeC32 | - | - | - | - | - | - | - | - | 0.32±0.21 | - |
| z-C33-1 | - | - | - | - | - | - | - | - | - | 0.69±0.31 |
| z-C33-2 | - | - | - | - | - | - | - | - | - | - |
| n-C33 | - | - | 0.06±0.04 | 0.15±0.06 | 0.09±0.17 | 0.58±0.81 | - | - | 0.24±0.03 | 0.30±0.23 |
| 17-;15-;13-MeC33 | - | - | - | 0.78±0.49 | 0.36±0.18 | 0.47±0.35 | - | - | - | - |
| 15-;13-;11-;9-MeC33 | - | - | - | - | - | - | - | - | - | - |
| 15-;13-;11-MeC33 | - | - | - | - | - | - | - | - | 11.08±0.79 | 11.35±2.48 |
| 15-; 13-MeC33 | 2.55±3.19 | 15.60b±5.49 | - | - | - | - | 3.76±0.27 | 3.19±0.75 | - | - |
| 13,17-; 11,15-diMeC33 | - | - | - | - | - | - | - | - | 7.38±0.92 | 7.04±1.76 |
| 13,17-diMeC33 | - | - | - | - | - | - | - | - | - | - |
| 13,15-; 11,13-diMeC33 | - | - | - | - | - | - | 3.02±0.37 | 2.25±0.79 | - | - |
| 7,X-diMeC33 | - | - | - | - | - | - | - | - | - | - |
| 3,15-; 3,13-diMeC33 | - | - | - | - | - | - | - | - | - | - |
| n-C34 | - | - | 0.04±0.06 | 0.21±0.76 | - | - | - | - | - | - |
| 17-;16-;14-;11-MeC34 | 2.78±4.18 | 2.19±0.82 | - | - | - | - | - | - | - | - |
| 17-;15-;13-MeC34 | - | - | - | 1.03±2.11 | - | - | - | - | - | - |
| 15-;14-;13-;12-MeC34 | - | - | - | - | - | - | - | - | - | - |
| 12,16-diMeC34 | - | - | - | - | - | - | - | - | - | - |
| 8,16-; 8,12-diMeC34 | - | - | - | - | - | - | - | - | - | - |
| x,y-diMeC34 - 1 | - | - | - | - | - | - | - | - | - | - |
| x,y-diMeC34 - 2 | - | - | - | - | - | - | - | - | - | - |
| n-C35 | - | - | 0.18±0.14 | 0.31±0.60 | - | - | - | - | - | - |
| 17-,15-,13-,11-,9-;7-MeC35 | - | - | - | - | - | - | - | - | - | - |
| 17-;15-;13-;11-MeC35 | - | - | 1.25±0.05 | 1.64±0.79 | - | - | - | - | 3.09±0.53 | 2.68±2.16 |
| 17-; 15-; 13-MeC35 | - | - | - | - | - | - | 1.43±0.34 | 1.18±0.55 | - | - |
| 13-MeC35 | 0.98±1.59 | 5.37±2.03 | - | - | - | - | - | - | - | - |
| 15,19-; 13,17-diMeC35 | - | - | - | - | - | - | - | - | - | - |
| 13,19-; 13,17-diMeC35 | - | - | - | - | - | - | - | - | 3.21±0.53 | 2.73±1.20 |
| 13,17-diMeC35 | - | - | 0.67±0.03 | 0.23±0.07 | - | - | - | - | - | - |
| 11,19-diMeC35 | - | - | 0.63±0.04 | - | - | - | - | - | - | - |
| 9,19-; 9,17-diMeC35 | - | - | 0.54±0.03 | - | - | - | - | - | - | - |
| 7,21-; 7,19-diMeC35 | - | - | 0.16±0.04 | - | - | - | - | - | - | - |
| 7,15-; 7,13-diMeC35 | - | - | - | - | - | - | - | - | - | - |
| 7,11-diMeC35 | - | - | - | - | - | - | - | - | - | - |
| 5,19-; 5,17-diMeC35 | - | - | 0.68±0.10 | - | - | - | - | - | - | - |
| 5,15-diMeC35 | - | - | - | - | - | - | - | - | - | - |
| 3,17-; 3,13-diMeC35 | - | - | - | - | - | - | - | - | - | - |
| 3,15-diMeC35 | - | - | - | - | - | - | - | - | - | - |
| 3,11-; 3,9-diMeC35 | - | - | - | - | - | - | - | - | - | - |
| 17-;15-;13-MeC36 | - | - | - | - | - | - | - | - | - | - |
| 10,16-; 10,14-diMeC36 | - | - | 0.36±0.09 | - | - | - | - | - | - | - |
| 8,16-; 8,12-diMeC36 | - | - | - | - | - | - | - | - | - | - |
| 3,17-; 3,11-diMeC36 | - | - | - | - | - | - | - | - | - | - |
| n-C37 | - | - | 0.71±0.09 | 0.08 | - | - | - | - | - | - |
| 17-;15-;13-MeC37 | - | - | 4.21±1.03 | 2.81±2.19 | - | - | - | - | - | - |
| 15-;13-;11-;9-;7-MeC37 | - | - | - | - | - | - | - | - | - | - |
| 15,19-; 13,19-MeC37 | - | - | 1.68±0.40 | 0.46±1.35 | - | - | - | - | - | - |
| 13,21-; 13,19-; 11,15-diMeC37 | - | - | - | - | - | - | - | - | - | - |
| 11,21-; 11,19-diMeC37 | - | - | 11.16±2.05 | 1.43±0.36 | - | - | - | - | - | - |
| 7,17-; 7,15-; 7,13-diMeC37 | - | - | - | - | - | - | - | - | - | - |
| 5,19-; 5,17-diMeC37 | - | - | 0.92±0.69 | 0.14±0.08 | - | - | - | - | - | - |
| x,y-diMeC37 | - | - | - | - | - | - | - | - | - | - |
| 3,x-diMeC37 | - | - | - | - | - | - | - | - | - | - |
| x,y,z-triMeC37-1 | - | - | - | 1.43±2.74 | - | - | - | - | - | - |
| x,y,z-triMeC37-2 | - | - | 1.40±0.22 | - | - | - | - | - | - | - |
| x,y,z-triMeC37-3 | - | - | 1.15±0.36 | - | - | - | - | - | - | - |
| x,y,z-triMeC37-4 | - | - | 2.01±0.12 | - | - | - | - | - | - | - |
| 17-; 13-; 11-MeC38 | - | - | - | - | - | - | 17.56±2.62 | 14.46±5.14 | - | - |
| 14-;12-MeC38 | - | - | - | - | - | - | - | - | - | - |
| n-C39 | - | - | 0.45±0.16 | - | - | - | - | - | - | - |
| 17-;15-MeC39 | - | - | 2.82±0.24 | 0.76±0.35 | - | - | - | - | - | - |
| 15-;13-;11-MeC39 | - | - | - | - | - | - | - | - | - | - |
| 13,15-; 11,13-diMeC39 | - | - | - | - | - | - | 1.63±0.44 | 1.60±0.37 | - | - |
| 11,21-; 11,19-diMeC39 | - | - | 24.69±1.42 | 1.90±0.77 | - | - | - | - | - | - |
| 13,X-diMeC39 | - | - | - | - | - | - | - | - | - | - |
| 11,x-diMeC39 | - | - | - | - | - | - | - | - | - | - |
| x,y-diMeC39-1 | - | - | - | 0.25±0.25 | - | - | - | - | - | - |
| 5,19-; 5,17-diMeC39 | - | - | 3.96±0.22 | - | - | - | - | - | - | - |
| x,y,z-triMeC39-1 | - | - | 0.28±0.19 | - | - | - | - | - | - | - |
| x,y,z-triMeC39-2 | - | - | 2.26±0.06 | - | - | - | - | - | - | - |
| 13-MeC40 | - | - | - | - | - | - | 2.10±0.55 | 3.76±1.29 | - | - |
| 11-MeC40 | - | - | - | - | - | - | 31.54±4.18 | 26.36±6.94 | - | - |
