# Supplementary material for: Cuticular hydrocarbons as caste-linked cues in Neotropical swarm-founding wasps
Source: PeerJ. 2022 Jun 7;10:e13571. doi: 10.7717/peerj.13571 (PMC9186331; doi:10.7717/peerj.13571)
Supplement: Supplemental Information 2 — Queens and workers of the remaining species shared the same CHC composition. [file peerj-10-13571-s002.docx]

Table S2: Cuticular hydrocarbons (CHCs) that were found exclusively in queens or workers in the epiponine wasp species. Queens and workers of the remaining species shared the same CHC composition.

| Species | *Clypearia sulcata* | | *Polybia paulista* | | *Charterginus* sp. | | *Agelaia pallipes* | | *Apoica pallens* | |
| --- | --- | --- | --- | --- | --- | --- | --- | --- | --- | --- |
| Compounds | queens | workers | queens | workers | queens | workers | queens | workers | queens | workers |
| n-C22 | - | - | - | 0.12±0.27 | - | - | - | - | - | - |
| z-C23-1 | - | - | 0.48±0.43 | - | - | - | - | - | - | - |
| z-C23-3 | - | - | - | - | - | 0.27±0.46 | - | - | - | - |
| *n*-C23 | - | - | - | - | - | - | 0.65±0.25 | - | - | - |
| *n*-C24 | - | 0.02±0.02 | - | - | - | - | - | - | - | - |
| *n*-C25 | - | 0.19±0.30 | - | - | - | - | - | - | - | - |
| 13-;11-MeC25 | - | - | - | 1.00±0.75 | - | - | - | - | - | - |
| 5-MeC25 | - | - | - | 0.23±0.28 | - | - | - | - | - | - |
| z-C27-4 | - | - | - | - | - | - | - | 0.18±0.08 | - | - |
| 13-MeC27 | - | - | - | - | - | - | - | - | 0.06±0.12 | - |
| 9-MeC27 | - | 0.07±0.11 | - | - | - | - | - | - | - | - |
| x,y-diMeC27-2 | - | - | - | - | - | - | - | 0.20±0.07 | - | - |
| 4-MeC28 | - | - | - | - | - | - | 0.58±0.13 | - | - | - |
| 3-MeC28 | - | - | - | - | - | 0.14±0.07 | - | - | - | - |
| 9-MeC29 | - | 0.03±0.03 | - | - | - | - | - | - | - | - |
| 7-MeC29 | - | 0.05±0.05 | - | 0.04±0.12 | - | - | - | - | - | - |
| 4-MeC29 | - | - | 0.17±0.16 | - | - | - | - | - | - | - |
| 3,13-diMeC29 | - | - | - | - | - | - | - | 0.31±0.46 | - | - |
| 15-;14-;13-MeC30 | - | 0.13±0.18 | - | - | - | - | - | - | - | - |
| 5,13-diMeC30 | - | - | - | - | - | - | 0.68±0.10 | - | - | - |
| x,y-diMeC30-1 | - | - | - | - | - | - | - | 1.12±0.38 | - | - |
| z-C31-5 | - | - | - | - | - | - | - | 0.44±0.17 | - | - |
| z-C31-9 | - | - | - | - | - | 0.18±0.24 | - | - | - | - |
| 7-MeC31 | - | 0.03±0.02 | - | - | - | - | - | - | - | - |
| 3-MeC31 | - | - | - | - | - | - | 0.17±0.10 | - | - | - |
| n-C32 | - | 0.07±0.07 | - | - | - | - | - | - | - | - |
| 16-;15-;14-;13-MeC32 | - | - | 0.12±0.12 | - | - | - | - | - | - | - |
| 13-;12-;11-MeC32 | - | 0.28±0.30 | - | - | - | - | - | - | - | - |
| 4-MeC32 | - | - | - | - | - | - | 0.32±0.21 | - | - | - |
| z-C33-1 | - | - | - | - | - | - | - | 0.69±0.31 | - | - |
| 17-;15-;13-MeC33 | - | - | - | - | - | 0.78±0.49 | - | - | - | - |
| n-C34 | - | 0.17±0.06 | - | - | - | - | - | - | - | - |
| 17-;15-;13-MeC34 | - | - | - | - | - | 1.03±2.11 | - | - | - | - |
| 8,16-; 8,12-diMeC34 | 0.08±0.04 | - | - | - | - | - | - | - | - | - |
| x,y-diMeC34 - 1 | 0.09±0.02 | - | - | - | - | - | - | - | - | - |
| x,y-diMeC34 - 2 | 0.10±0.01 | - | - | - | - | - | - | - | - | - |
| 11,19-diMeC35 | - | - | - | - | 0.63±0.04 | - | - | - | - | - |
| 9,19-; 9,17-diMeC35 | - | - | - | - | 0.54±0.03 | - | - | - | - | - |
| 7,21-; 7,19-diMeC35 | - | - | - | - | 0.16±0.04 | - | - | - | - | - |
| 7,15-; 7,13-diMeC35 | - | 1.08±0.52 | - | - | - | - | - | - | - | - |
| 7,11-diMeC35 | 1.60±0.15 | - | - | - |  | - | - | - | - | - |
| 5,19-; 5,17-diMeC35 | - | - | - | - | 0.68±0.10 | - | - | - | - | - |
| 3,17-; 3,13-diMeC35 | - | 1.00±0.26 | - | - | - | - | - | - | - | - |
| 3,15-diMeC35 | 1.23±0.09 | - | - | - | - | - | - | - | - | - |
| 10,16-; 10,14-diMeC36 | - | - | - | - | 0.36±0.09 | - | - | - | - | - |
| 3,17-; 3,11-diMeC36 | 0.25±0.06 | - | - | - | - | - | - | - | - | - |
| x,y-diMeC37 | - | 1.59±0.75 | - | - | - | - | - | - | - | - |
| 3,x-diMeC37 | 1.15±0.08 | - | - | - | - | - | - | - | - | - |
| x,y,z-triMeC37-1 | - | - | - | - | - | 1.43±2.74 | - | - | - | - |
| x,y,z-triMeC37-2 | - | - | - | - | 1.40±0.22 | - | - | - | - | - |
| x,y,z-triMeC37-3 | - | - | - | - | 1.15±0.36 | - | - | - | - | - |
| x,y,z-triMeC37-4 | - | - | - | - | 2.01±0.12 | - | - | - | - | - |
| n-C39 | - | - | - | - | 0.45±0.16 | - | - | - | - | - |
| 13,X-diMeC39 | - | 0.67±0.43 | - | - | - | - | - | - | - | - |
| 11,x-diMeC39 | 0.21±0.06 | - | - | - | - | - | - | - | - | - |
| x,y-diMeC39-1 | - | - | - | - | - | 0.25±0.25 | - | - | - | - |
| 5,19-; 5,17-diMeC39 | - | - | - | - | 3.96±0.22 | - | - | - | - | - |
| x,y,z-triMeC39-1 | - | - | - | - | 0.28±0.19 | - | - | - | - | - |
| x,y,z-triMeC39-2 | - | - | - | - | 2.26±0.06 | - | - | - | - | - |
